# HAP40 modulates mutant Huntingtin aggregation and toxicity in Huntington’s disease mice

**DOI:** 10.1038/s41419-024-06716-4

**Published:** 2024-05-14

**Authors:** Laiqiang Chen, Yiyang Qin, Tingting Guo, Wenzhen Zhu, Jingpan Lin, Tingting Xing, Xuezhi Duan, Yiran Zhang, Eshu Ruan, Xiang Li, Peng Yin, Shihua Li, Xiao-Jiang Li, Su Yang

**Affiliations:** 1 Guangdong Key Laboratory of Non-human Primate Research, Key Laboratory of CNS Regeneration (Ministry of Education), Guangdong-Hongkong-Macau Institute of CNS Regeneration, Jinan University, Guangzhou, China; 2 State Key Laboratory of Bioactive Molecules and Druggability Assessment, Jinan University, Guangzhou, China; 3 Department of Medical Genetics and Cell Biology, School of Basic Medical Sciences, Jiangxi Medical College, Nanchang University, Nanchang, China

**Keywords:** Huntington's disease, Protein aggregation

## Abstract

Huntington’s disease (HD) is a monogenic neurodegenerative disease, caused by the CAG trinucleotide repeat expansion in exon 1 of the Huntingtin (HTT) gene. The *HTT* gene encodes a large protein known to interact with many proteins. Huntingtin-associated protein 40 (HAP40) is one that shows high binding affinity with HTT and functions to maintain HTT conformation in vitro. However, the potential role of HAP40 in HD pathogenesis remains unknown. In this study, we found that the expression level of HAP40 is in parallel with HTT but inversely correlates with mutant HTT aggregates in mouse brains. Depletion of endogenous HAP40 in the striatum of HD140Q knock-in (KI) mice leads to enhanced mutant HTT aggregation and neuronal loss. Consistently, overexpression of HAP40 in the striatum of HD140Q KI mice reduced mutant HTT aggregation and ameliorated the behavioral deficits. Mechanistically, HAP40 preferentially binds to mutant HTT and promotes Lysine 48-linked ubiquitination of mutant HTT. Our results revealed that HAP40 is an important regulator of HTT protein homeostasis in vivo and hinted at HAP40 as a therapeutic target in HD treatment.

## Introduction

Huntington’s disease (HD) is an autosomal dominant neurodegenerative disease [1]. The causative gene for HD is *Huntingtin (HTT)*, which carries CAG trinucleotide repeats that are translated into a polyglutamine (polyQ) tract. Over 36 CAG repeats are considered mutant, which leads to mutant HTT (mHTT) toxicity and disease onset in HD patients [2, 3]. HD is characterized by preferential neuronal loss in the striatum [4]. As the disease progresses, degeneration also occurs in other brain regions, whereas the cerebellum is less affected [5–8].

HTT is a large protein composed of the N-terminal HEAT (Huntingtin, elongation factor 3, protein phosphatase 2A, and the yeast kinase TOR1) domain, intermediate bridge domain, and C-terminal HEAT domain [9, 10], and is believed to function as a scaffold-like protein that is involved in multiple biological processes including signal transduction, cell proliferation, autophagy, and cell differentiation [11–14]. The full-length HTT protein undergoes cleavage by multiple proteases including caspases and calpains [15–18]. The N-terminal mHTT fragments that harbor the expanded polyQ tract are prone to aggregation and are the predominant components of the inclusion bodies in the HD patient brains [16, 19, 20]. Although the exact role of mHTT aggregates in HD pathogenesis remains elusive, it is generally accepted that the formation of mHTT aggregates reflects important pathological processes, and most HD therapies tested so far are accompanied by a reduction of mHTT aggregation [21–24]. The ubiquitin-proteasome system (UPS) and autophagy are two major degradation pathways for mHTT, which reduce the load of misfolded mHTT and protect against mHTT-induced neurotoxicity [25–27].

HTT is known to interact with numerous proteins [28–30]. Among such proteins, Huntingtin-associated protein 40 (HAP40) shows a strong and stable interaction with HTT [31], and the expression levels of HTT or HAP40 appear to be dependent on each other [32, 33]. Furthermore, the conformation of HTT protein is stabilized upon HAP40 binding, so that its structure could be determined by cryo-electron microscopy [9]. These results indicate that HAP40 could play a role in modulating mHTT dynamics and HD pathogenesis. However, studies based on cellular models yielded conflicting results, as HAP40 has been shown to enhance or inhibit mHTT toxicity [34, 35]. Therefore, how HAP40 affects mHTT turnover in vivo remains a matter of debate.

In this study, we utilized a widely used knock-in mouse model of HD (HD140Q KI) that expresses full-length mHTT at an endogenous level [36] to investigate the role of HAP40 in HD pathogenesis and the underlying mechanism. By comparing protein expression in different brain regions of HD140Q KI mice, we found that HAP40 positively correlates with full-length WT HTT and mHTT, but negatively correlates with insoluble mHTT aggregates. Increasing and decreasing HAP40 expression in the striatum of HD140Q KI mice via viral injection inhibits and enhances mHTT aggregation and HD-like phenotypes, respectively. Mechanistically, HAP40 preferentially binds to mHTT and truncated N-terminal mHTT fragments and promotes Lysine 48 (K48)-linked ubiquitination of mHTT, which is a well-established signal for proteasomal degradation. Overall, our study revealed HAP40 as a critical regulator of mHTT aggregation in the mammalian brain and hinted a promising therapeutic target for future HD treatment.

## Results

### Characterization of HAP40 and HTT expression in the mouse brain

**Fig. 1:**
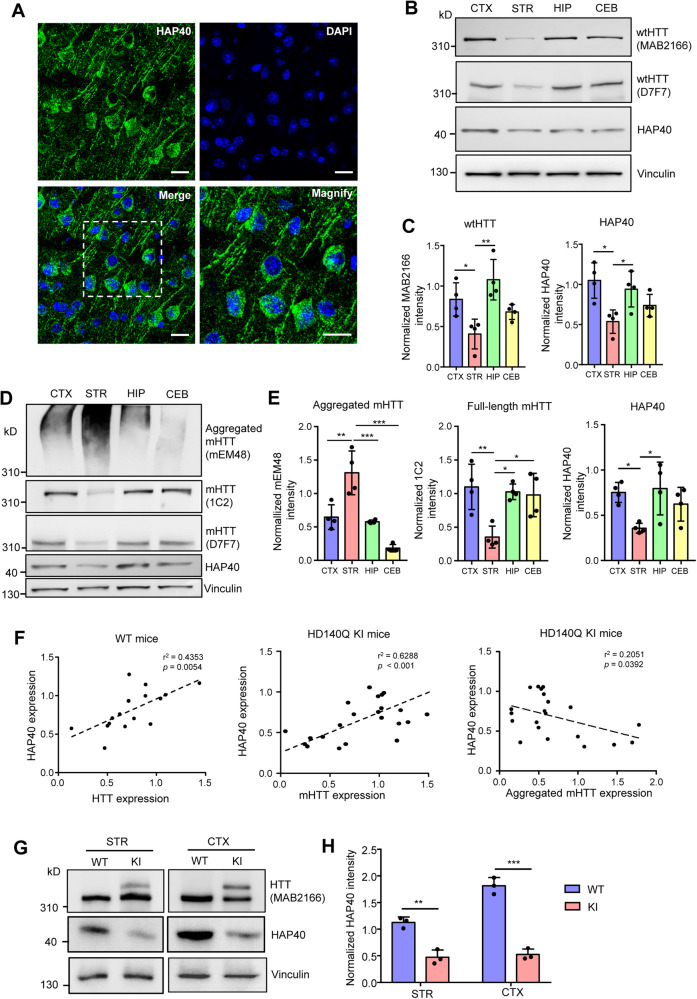
Characterization of HAP40 expression in the mouse brain. **A** Immunofluorescent staining of the cortex slices showed that HAP40 is predominately localized in the cytoplasm (40×, scale bar: 20 μm). **B** Western blotting analysis of wild-type HTT (WT HTT, antibody MAB2166, and D7F7) and HAP40 expression in different brain regions of wild-type mice (CTX cortex, STR striatum, HIP hippocampus, CEB cerebellum). **C** Quantitative results of WT HTT and HAP40 expression in different brain regions (*n* = 4, one-way ANOVA with Tukey post-tests, WT HTT, CTX vs STR, *P* = 0.0360, STR vs HIP, *P* = 0.0016; HAP40, CTX vs STR, *P* = 0.0101, STR vs HIP, *P* = 0.0427). **D** Western blotting analysis of full-length mutant HTT (mHTT, antibody 1C2 and D7F7) and mutant HTT aggregates (Aggregated mHTT, antibody mEM48) in different brain regions of homozygous HD140Q KI mice. **E** Quantitative results of mHTT aggregates, full-length mHTT, and HAP40 expression in different brain regions (*n* = 4, one-way ANOVA with Tukey post-tests, aggregated mHTT, CTX vs STR, *P* = 0.0017, STR vs HIP, *P* = 0.0007, STR vs CEB, *P* < 0.0001; full-length mHTT, CTX vs STR, *P* = 0.0062, STR vs HIP, *P* = 0.0127, STR vs CEB, *P* = 0.0200; HAP40, CTX vs STR, *P* = 0.0440, STR vs HIP, *P* = 0.0247). **F** Linear regression analysis correlating HAP40 level with the expression of WT HTT, full-length mHTT, and aggregated mHTT in different brain regions of WT and HD140Q KI mice (*n* = 16–24). **G** Western blot analysis of HAP40 and HTT expression in the cortex and striatum of heterozygous HD140Q KI and WT mice. **H** Quantitative results of HAP40 expression in heterozygous HD140Q KI and WT mice (*n* = 3, two-tailed student *t*-test, STR, *P* = 0.0018; CTX, *P* < 0.0001). *P < 0.05; **P < 0.01; ***P < 0.001. Data are presented as mean values ± SEM.

We examined the expression of HAP40 in the adult mouse brain via immunohistochemistry. The specificity of the HAP40 antibody has been confirmed by western blotting in the murine N2A cells with *Hap40* knockdown via siRNAs (Fig. S1A). HAP40 is expressed throughout the brain. High-magnification images of the cortex and striatum revealed that HAP40 is mainly localized in the cytoplasm, and some cells displayed dot-like structures in the nucleus (Fig. 1A, Fig. S1B). This result is further confirmed by western blotting analysis, as more HAP40 is found in the cytoplasmic fraction compared with the nuclear fraction (Fig. S1C). We also performed double staining of HAP40 with neuronal markers including NeuN and β3-tubulin, astrocyte marker GFAP, or microglia marker F4/80. We found that the HAP40 signal overlapped with NeuN and β3-tubulin, but barely with GFAP or F4/80, suggesting that HAP40 is preferentially expressed in the neurons, but not in the glial cells (Fig. S2A). In addition, we dissected mouse cortical gray matter, which is enriched with neurons, and white matter, which is enriched with glial cells. Western blotting analysis showed that more HAP40 is found in the gray matter (Fig. S2B), which is consistent with the double staining results.

Previous studies indicate that HAP40 and HTT stabilize each other in cultured cells [32]. To investigate the relationship between HAP40 and HTT in vivo, we collected brain tissues from 4-month-old wild-type (WT) mice and examined the expression of HTT and HAP40 via western blotting. The cortex, striatum, hippocampus, and cerebellum were dissected to study brain regions that are differentially affected in HD. We used several HTT antibodies that have been verified to recognize specific forms of HTT proteins. For example, the mEM48 antibody is good for detecting mHTT aggregates, the MAB2166 and D7F7 antibodies recognize both soluble full-length WT and mHTT, and the 1C2 antibody preferentially recognizes full-length mHTT [37–40]. The levels of HTT and HAP40 were in parallel in different brain regions of WT mice, with higher expression in the cortex and hippocampus and lower expression in the striatum and cerebellum (Fig. 1B, C). We repeated this experiment using brain tissues of 10-month-old homozygous HD140Q KI mice in which aggregated mHTT becomes visible and found a similar expression pattern between HAP40 and full-length mHTT. Nonetheless, an inverse correlation between HAP40 and aggregated mHTT was detected (Fig. 1D, E), as the striatal tissue showed the highest level of mHTT aggregates and the lowest level of HAP40. Linear regression analysis further indicated a positive correlation between HAP40 and full-length WT HTT/mHTT, and a negative correlation between HAP40 and mHTT aggregates (Fig. 1F). We also measured the mRNA level of *Htt* and *Hap40* in the brain of WT mice via quantitative real-time PCR. The mRNA level of *Htt* and *Hap40* did not correlate with their protein level, suggesting that the rate of HTT and HAP40 synthesis or degradation varies among different brain regions. Nonetheless, *Htt* and *Hap40* still showed a comparable pattern of mRNA level in the cortex, striatum, hippocampus, and cerebellum (Fig. S3A, B). In addition, the expression of HAP40 was significantly reduced in the cortex and striatum of heterozygous HD140Q KI mice, compared with age-matched WT mice (Fig. 1G, H), but the mRNA levels of *Hap40* are similar when comparing HD140Q KI and WT mice (Fig. S3C).

### Knocking down *Hap40* enhanced mHTT aggregation in HD140Q KI mice

To verify that HAP40 regulates the turnover of mHTT in vivo, we constructed gRNA plasmids targeting *Hap40* gene, so that HAP40 expression can be reduced using CRISPR/Cas9-mediated genome editing. All three gRNAs designed (#2, #50, #93) showed DNA cutting capacity via T7E1 assay when tested in N2A cell line (Fig. S4A). We packaged two gRNA plasmids (#2 and #50) into adeno-associated virus (AAV), mixed with AAV-Cas9, and delivered into the striatum of WT mice via stereotaxic injection. By western blotting analysis, we found that #50 gRNA led to a more reduction of HAP40 (Fig. S4B) so that AAV expressing #50 gRNA (AAV-Hap40-gRNA) was used in the following knockdown experiments.

**Fig. 2:**
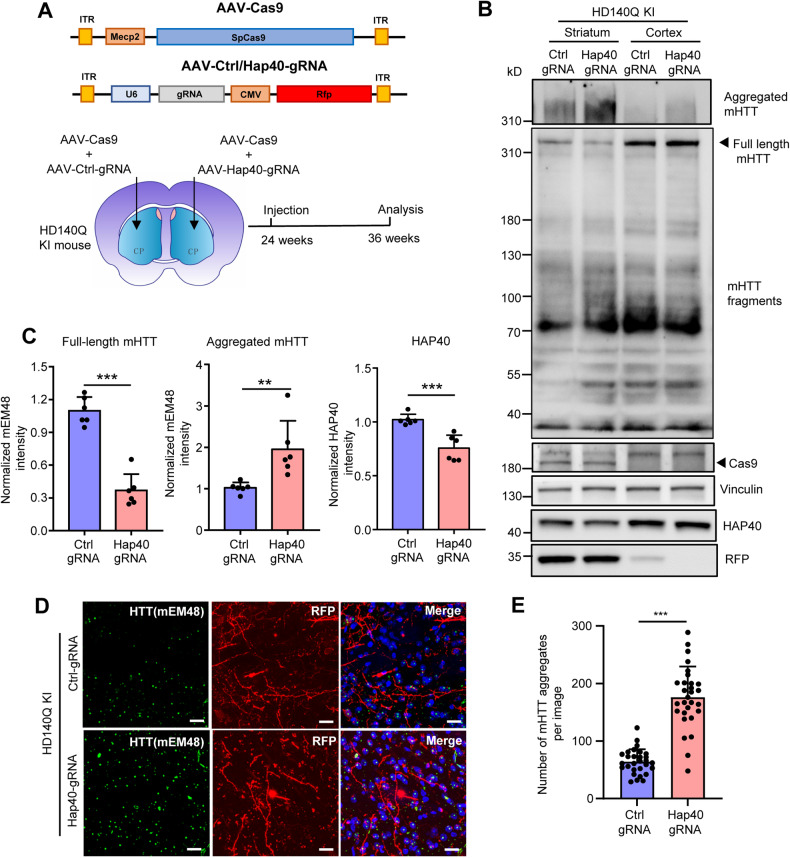
Reducing HAP40 expression enhances mHTT aggregation. **A** A schematic representation of the CRISPR/Cas9 viruses and the injection sites in the brain. Heterozygous HD140Q KI mice were injected with AAV-Cas9/AAV-Ctrl-gRNA in the left striatum, and with AAV-Cas9/AAV-Hap40-gRNA in the right striatum. The mice received injection at the age of 24 weeks and were euthanized at the age of 36 weeks. **B** Western blotting analysis of the cortex and striatum tissues from HD140Q KI mice 12 weeks after virus injection in the striatum. The cortex tissues were not injected with AAVs, but had some virus expression caused by AAVs spreading from the striatum. Full-length mHTT, mHTT fragments, and mHTT aggregates were revealed by the mEM48 antibody. Cas9 and RFP antibodies were used to confirm viral expression in the striatum. Vinculin served as a loading control. **C** Quantitative results of full-length mHTT, aggregated mHTT, and HAP40 expression in the injected striatal tissues (*n* = 6, two-tailed student *t*-test, full-length mHTT, *P* < 0.0001; aggregated mHTT, *P* = 0.0087; HAP40, *P* = 0.0006). **D** Immunofluorescent staining showed that the number of mHTT aggregates (detected by mEM48 antibody) were increased in the striatal areas with HAP40 knockdown (40×, scale bar: 20 μm). **E** Quantitative results of the number of mHTT aggregates in the immunofluorescent staining images (*n* = 30 from three mice, two-tailed student *t*-test, *P* < 0.0001). **P < 0.01; ***P < 0.001. Data are presented as mean values ± SEM.

We mixed AAV-Cas9 with either AAV-Control-gRNA or AAV-Hap40-gRNA, and unilaterally injected the viruses into the striatum of 6-month-old heterozygous HD140Q KI mice (Fig. 2A). Twelve weeks later, the mice were sacrificed, and the brain tissues were analyzed by western blotting. The AAV-Cas9/AAV-Hap40-gRNA viruses led to a ~30% reduction of HAP40 expression in the striatum, which was accompanied by a significant decrease of full-length mHTT and an increase of mHTT aggregates (Fig. 2B, C). This result is further supported by immunofluorescent staining. As the AAV-Hap40-gRNA also expresses RFP, we found more mHTT aggregates stained by the mEM48 antibody in the area expressing AAV-Cas9/AAV-Hap40-gRNA (Fig. 2D, E).

### Broad HAP40 reduction caused severe neurodegeneration in HD140Q KI mice

**Fig. 3:**
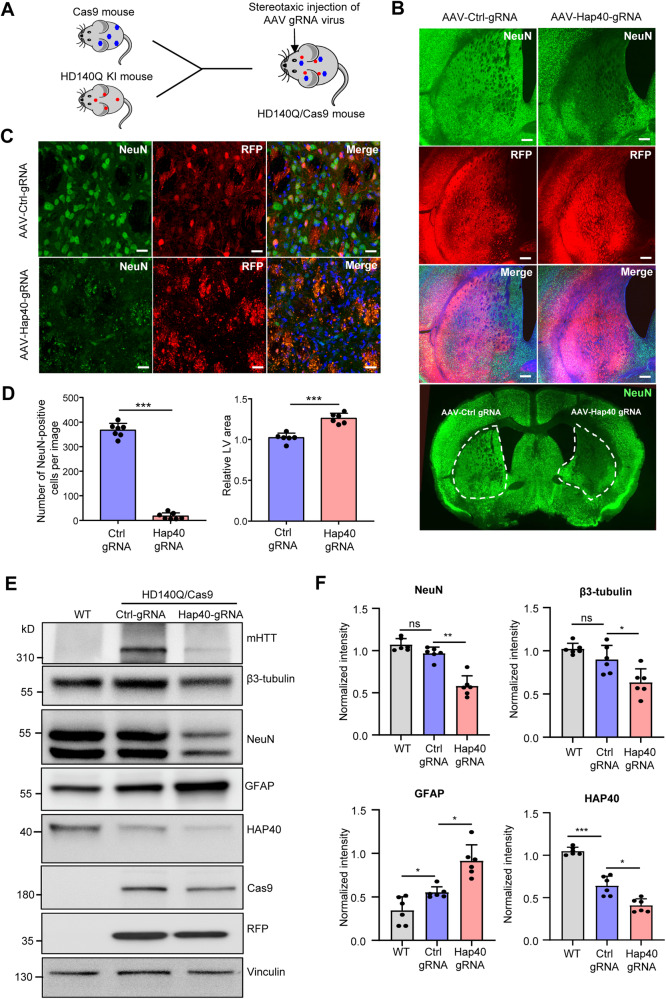
Reducing HAP40 expression led to severe neurodegeneration in HD140Q/Cas9 mice. **A** The germline Cas9 mice were crossed with heterozygous HD140Q KI mice to generate HD140Q/Cas9 mice. HD140Q/Cas9 mice at the age of 6 months were injected with AAV-Ctrl-gRNA in the left striatum and with AAV-Hap40-gRNA in the right striatum. **B** One month after viral injection, immunofluorescent staining showed that the NeuN signal was dramatically decreased in the AAV-Hap40-gRNA injected region, compared to the AAV-Ctrl-gRNA injected region in the striatum (1×, scale bar: 200 μm). **C** High-magnification images of NeuN staining in the striatal slices injected with AAV-Hap40-gRNA or AAV-Ctrl-gRNA (40×, scale bar: 20 μm). **D** Quantitative results of the number of NeuN-positive cells and the relative lateral ventricle (LV) area in the immunofluorescent staining images (*n* = 6–7, two-tailed student *t*-test, NeuN, *P* < 0.0001; LV area, *P* < 0.0001). **E** Western blotting analysis of the striatum tissues from WT mice and HD140Q/Cas9 mice one month after viral injection. Full-length mHTT was revealed by the 1C2 antibody. NeuN, β3-tubulin, and GFAP antibodies were used to indicate neuronal damage. Vinculin was used as a loading control. **F** Quantitative results of NeuN, β3-tubulin, GFAP, and HAP40 expression in the injected striatal tissues (*n* = 6, one-way ANOVA with Tukey post-tests; NeuN, *P* = 0.0023; β3-tubulin, *P* = 0.0347; GFAP, WT vs Ctrl gRNA, *P* = 0.0297, Ctrl gRNA vs Hap40 gRNA, *P* = 0.0246; HAP40, WT vs Ctrl gRNA, *P* = 0.0008, Ctrl gRNA vs Hap40 gRNA, *P* = 0.0104). Ns not significant; *P < 0.05; **P < 0.01; ***P < 0.001. Data are presented as mean values ± SEM.

As the size of Cas9 cDNA is very large, AAV-Cas9 transduction is not very efficient and can only infect limited brain areas. To ensure the knockdown of *Hap40* in the entire striatum, we crossed heterozygous HD140Q KI mice with germline Cas9 mice [41], so that the derived HD140Q/Cas9 mice express mHTT and Cas9 throughout the body (Fig. 3A). HD140Q/Cas9 mice at the age of 6 months were injected with AAV-Control-gRNA in one side of the striatum, and AAV-Hap40-gRNA in the other side. One month after injection, we saw a dramatic reduction of NeuN, DARPP32, and β3-tubulin positive signals on the side expressing AAV-Hap40-gRNA (Fig. 3B, C, Fig. S5A, B), suggesting a massive loss of neurons. In contrast, the GFAP staining intensity was increased, which is indicative of astrocyte reactivity (Fig. S5C). The significant reduction of NeuN-positive cells in the striatum was accompanied by a significantly enlarged lateral ventricle (Fig. 3D). Immunohistochemistry showed that *Hap40* knockdown led to more mHTT aggregates in the striatum (Fig. S5D, E). We also performed western blotting to compare the extent of neuronal loss in the striatum of WT mice, and HD140Q/Cas9 mice injected with AAV-Control-gRNA or AAV-Hap40-gRNA. Although HAP40 expression was already reduced in HD140Q/Cas9 mice injected with AAV-Control-gRNA, obvious neuronal damage was only seen in HD140Q/Cas9 mice injected with AAV-Hap40-gRNA, indicated by the significant reduction of NeuN and β3-tubulin, and significant increase of GFAP (Fig. 3E, F). This result is consistent with previous findings that no obvious neurodegeneration was found in HD140Q KI mice until at very late stages [21, 36, 40, 42], and also suggests that the expression of HAP40 needs to drop below certain thresholds to enhance the neurotoxicity of mHTT.

To determine if HAP40 reduction causes similar neurodegeneration in WT mice, we injected the same virus into the striatum of 6-month-old germline Cas9 mice. We saw a concomitant reduction of WT HTT when *Hap40* was knocked down but did not find significant changes in NeuN or GFAP expression when comparing AAV-Hap40-gRNA and AAV-Control-gRNA injected tissues (Fig. S6A, B). This conclusion was further confirmed by immunofluorescent staining of the striatal slices, as the number of NeuN-positive cells was comparable between AAV-Hap40-gRNA and AAV-Control-gRNA-infected areas (Fig. S6C, D).

### Overexpression of HAP40 ameliorates mHTT aggregation and HD phenotypes

**Fig. 4:**
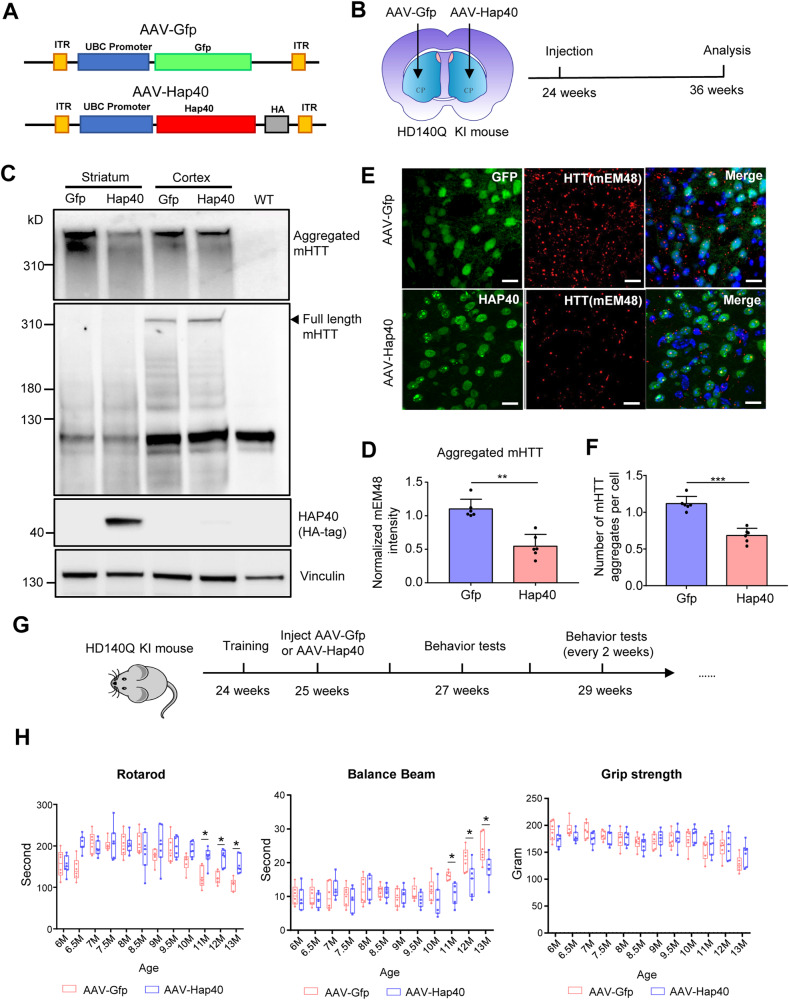
Overexpression of HAP40 attenuates mHTT aggregation and toxicity. **A** A schematic representation of the AAV-Gfp and AVV-Hap40 constructs. **B** Heterozygous HD140Q KI mice were injected with AAV-Gfp in the left striatum, and with AAV-Hap40 in the right striatum. **C** Western blotting analysis of full-length mHTT and aggregated mHTT in the striatum and cortex of HD140Q KI mice 12 weeks after viral injection. Vinculin was used as a loading control. The cortex tissues were not injected with AAVs and served as controls. The striatum tissue from a WT mouse was used to indicate mHTT-specific bands. **D** Quantitative results of aggregated mHTT in the injected striatum tissues (*n* = 6, two-tailed student *t*-test, *P* = 0.0031). **E** Immunofluorescent staining of mHTT aggregates (detected by mEM48 antibody) in the striatum of HD140Q KI mice injected with AAV-Gfp or AAV-Hap40 (40×, scale bar: 20 μm). **F** Quantitative results of the number of mHTT aggregates in the immunofluorescent staining images (*n* = 6, two-tailed student *t*-test, *P* < 0.0001). **G** Heterozygous HD140Q KI mice at the age of 25 weeks received bilateral injections of AAV-Gfp or AAV-Hap40 in the striatum. Their behavioral performances were examined every two weeks after the viral injection. **H** Compared to HD140Q KI mice injected with AAV-Gfp, HD140Q KI mice injected with AAV-Hap40 showed significant improvements in the rotarod and balance beam tests starting at 11 months of age (*n* = 6–7, two-way ANOVA with Sidak post-tests, rotarod, overall, *P* = 0.0033; 11 M, *P* = 0.0164; 12 M, *P* = 0.0189; 13 M, *P* = 0.0222; balance beam, overall, *P* = 0.0143; 11 M, *P* = 0.0428; 12 M, *P* = 0.0112; 13 M, *P* = 0.0171). *P < 0.05; **P < 0.01; ***P < 0.001. Data are presented as mean values ± SEM.

To test whether increasing HAP40 expression leads to the opposite effects, we constructed a plasmid encoding full-length mouse HAP40 fused with a C-terminal HA tag (AAV-Hap40) and packaged the plasmid into AAV9 viruses (Fig. 4A). AAV-Hap40 or AAV-Gfp as control was separately injected into one side of the striatum in 6-month-old heterozygous HD140Q KI mice, and the injected mice were kept for 12 weeks before evaluation (Fig. 4B). The expression of exogenous AAV-Hap40 in the striatum was confirmed by western blotting with an HA antibody. Importantly, compared with the side of the striatum injected with AAV-Gfp, the AAV-Hap40 injected side showed significantly reduced mHTT aggregation (Fig. 4C, D). As a control, the cortex tissues of both sides displayed comparable levels of mHTT aggregation, as the viral expression was restricted to the striatum. Immunofluorescent staining revealed that HAP40 overexpression reduced the number of mHTT aggregates (Fig. 4E, F). Interestingly, the exogenous HAP40 exhibited dot-like patterns and co-localized with mHTT aggregates in the nucleus of striatal neurons (Fig. S7A, B). We also injected AAV-Hap40 into the striatum of WT mice and found that the exogenous HAP40 was diffused in the nucleus of striatal neurons without dot-like staining (Fig. S7C). Additionally, the injection of AAV-Hap40 did not change the expression of WT HTT in the striatum of WT mice, nor did it change the expression of NeuN (Fig. S7D, E).

Previous studies have extensively characterized the behavioral performances of HD140Q KI mice compared to WT mice [21, 36, 40, 42]. The pathological phenotypes of HD140Q KI mice are generally mild, as they started to show motor deficits around 6–9 months of age compared to WT mice. Therefore, we focused on whether HAP40 overexpression can influence the motor functions of HD140Q KI mice. We bilaterally injected AAV-Hap40 into the striatum of 6-month-old heterozygous HD140Q KI mice and monitored their motor activities every two weeks (Fig. 4G). Compared with the control HD140Q KI mice injected with AAV-Gfp, the AAV-Hap40-injected mice started to show behavioral improvements at the age of 11 months, demonstrated by significantly better performances in both the rotarod and balance beam tests (Fig. 4H). The AAV-Hap40 injected mice also performed better in the grip strength test, though not significantly. The expression of AAV-Hap40 was confirmed in 13-month-old HD140Q KI mice, 7 months after the stereotaxic surgery (Fig. S8A).

### HAP40 preferentially binds mHTT and regulates its ubiquitination

**Fig. 5:**
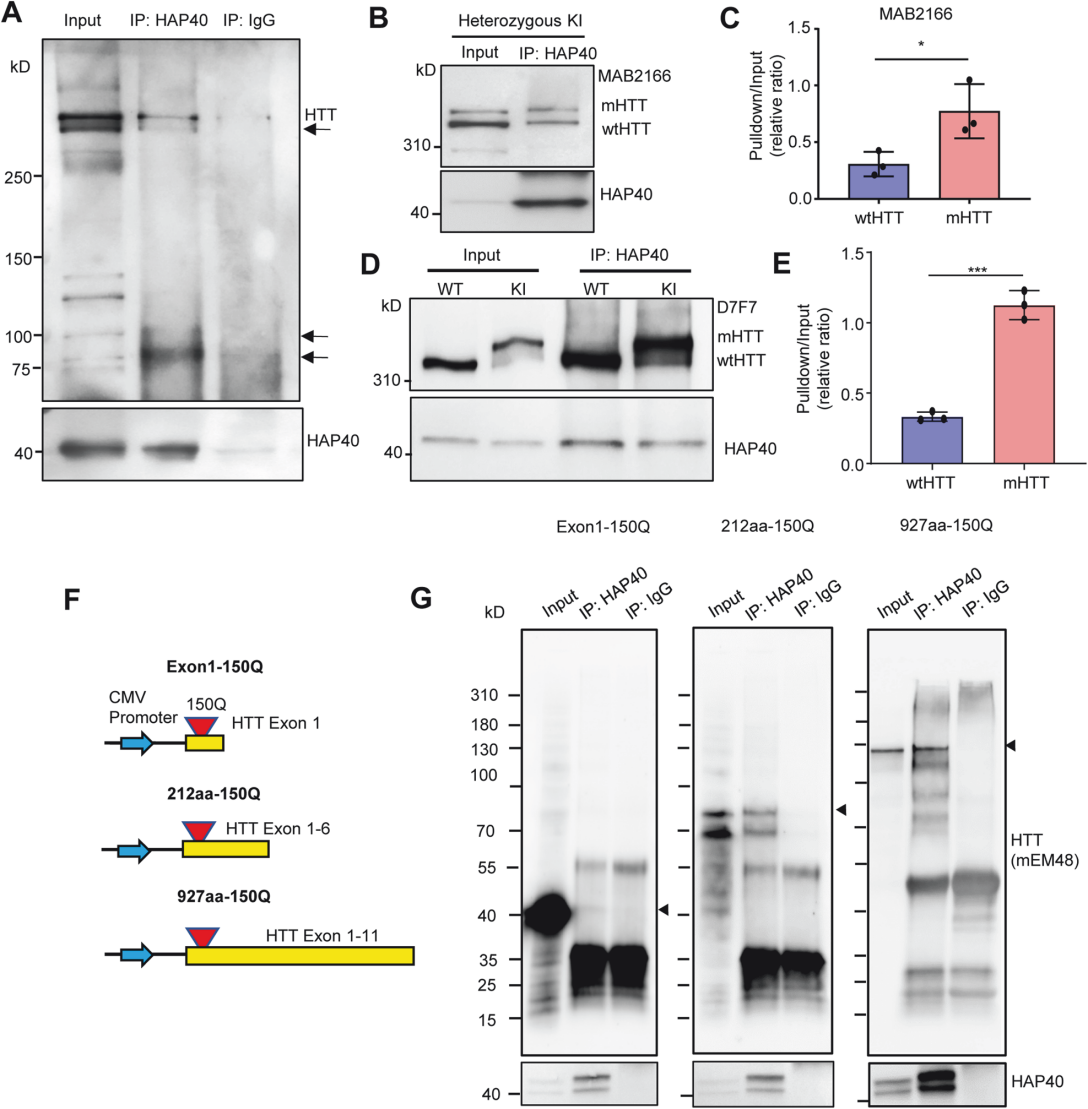
HAP40 interacts with both full-length mHTT and selective N-terminal mHTT fragments. **A** Co-immunoprecipitation assay was performed using the brain samples of heterozygous HD140Q KI mice. The HAP40 antibody was used to precipitate the endogenous HAP40 protein, and the mEM48 antibody was used to reveal both full-length mHTT and N-terminal mHTT fragments. Arrows indicate some mHTT fragments that were co-immunoprecipitated with HAP40. **B** Co-immunoprecipitation assay using the HAP40 antibody and heterozygous HD140Q KI brain lysate. The MAB2166 antibody was used to reveal both full-length WT HTT and mHTT in the precipitates. **C** Quantitative results of the relative ratio of WT HTT and mHTT pulled down by HAP40, detected by MAB2166 antibody (*n* = 3, two-tailed student t-test, *P* = 0.0369). **D** The co-immunoprecipitation result was repeated using another HTT antibody D7F7. **E** Quantitative results of the relative ratio of WT HTT and mHTT pulled down by HAP40, detected by D7F7 antibody (*n* = 3, two-tailed student *t*-test, *P* = 0.0002). **F** A schematic representation of plasmids expressing different N-terminal mHTT fragments. **G** Co-immunoprecipitation assay was performed using HEK293 cells transfected with the HAP40 plasmid and N-terminal mHTT of different lengths. The HAP40 antibody was used to pull down HAP40, and the mEM48 antibody was used to reveal different N-terminal mHTT fragments in the precipitates. *P < 0.05; ***P < 0.001. Data are presented as mean values ± SEM.

Mass spectrometry analysis has identified a large number of HTT-interacting proteins [30, 43]. Validation of these potential interacting proteins requires independent approaches, such as the commonly used co-immunoprecipitation assay [44–46], preferentially in a physiologically relevant setting. Therefore, we examined HAP40 and HTT interaction in vivo by co-immunoprecipitation, using the brain lysates of heterozygous HD140Q KI mice. By pulling down HAP40 and probing HTT with the mEM48 antibody that recognizes full-length mHTT and mHTT fragments, we found that both full-length mHTT and certain mHTT fragments were co-purified with HAP40 (Fig. 5A). Comparing the ratio of HTT in the pulldown lysate versus in the input on the same blot provides a stringent way to distinguish the binding affinity of HAP40 with WT HTT and mHTT. Using the MAB2166 antibody that preferentially recognizes WT HTT as compared to mHTT [21, 47], we detected a significantly higher ratio of mHTT than WT HTT in the precipitated lysate (Fig. 5B, C). We repeated the experiment using another HTT antibody D7F7, which has a stronger reaction to mHTT [39] (Fig. S8B, C), and again found that a higher ratio of mHTT was pulled down by HAP40 (Fig. 5D, E), therefore indicating that HAP40 preferentially binds to mHTT. To explore which N-terminal mHTT fragments interact with HAP40, we constructed plasmids expressing N-terminal mHTT of different sizes (exon 1 HTT, first 212, and 927 amino acids) (Fig. 5F). Each of these plasmids was transfected into HEK293 cells together with a plasmid expressing HAP40, and the cell lysate was used for co-immunoprecipitation assay. We found that HAP40 could precipitate mHTT fragments containing the first 212 and 927 amino acids, but not exon 1 mHTT (Fig. 5G). These results suggest that HAP40 binds to N-terminal mHTT fragments that are longer than exon 1 HTT.

**Fig. 6:**
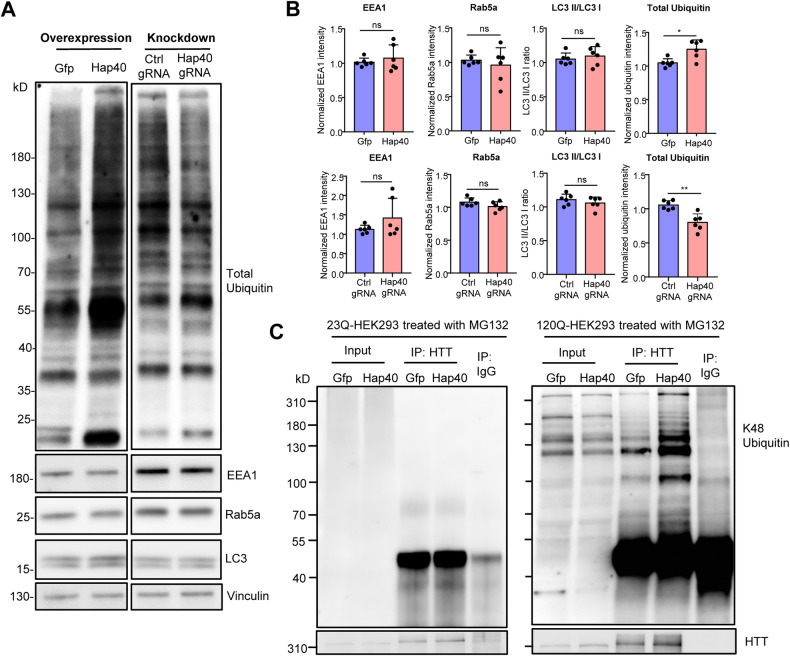
HAP40 promotes K48-linked ubiquitination of mHTT. **A** Western blotting analysis of total ubiquitin and autophagic markers (EEA1, Rab5a, and LC3) in the striatum tissues with HAP40 overexpression (Hap40) or knockdown (Hap40 gRNA). **B** Quantitative results of protein expression examined in the injected striatal tissues (*n* = 6, two-tailed student *t*-test, GFP vs HAP40: EEA1, *P* = 0.4546; Rab5a, *P* = 0.5523; LC3 II/LC3 I, *P* = 0.4148; total ubiquitin, *P* = 0.0110; Ctrl gRNA vs Hap40 gRNA: EEA1, *P* = 0.5975; Rab5a, *P* = 0.2219; LC3 II/LC3 I, *P* = 0.0551; total ubiquitin, *P* = 0.0053). Upper panel: the striatum tissues with HAP40 overexpression; lower panel: the striatum tissues with HAP40 knockdown. **C** HEK293 cells stably expressing WT HTT (23Q-HEK293) or mHTT (120Q-HEK293) were transfected with the Hap40 plasmid for 48 h, and then treated with MG132 for another 12 h. HTT was precipitated by the EPR5526 antibody and the K48 ubiquitin antibody was used to detect K48-linked ubiquitination. Cells transfected with the Gfp plasmid were used as a control. Ns, not significant; **P < 0.01; ***P < 0.001. Data are presented as mean values ± SEM.

The UPS and autophagy are the two major cellular mechanisms to clear misfolded proteins [48]. To investigate which pathway requires HAP40 in vivo, we examined the expression of major proteins related to the UPS and autophagy in the striatum of HD140Q KI mice injected with AAV-Hap40 to overexpress HAP40 or injected with AAV-Cas9/AAV-Hap40-gRNA to reduce HAP40 expression. We did not detect any significant changes in the expression of EEA1, Rab5a, or LC3 (Fig. 6A, B), suggesting that HAP40 expression changes are not likely to alter the autophagy activity. In contrast, the total ubiquitin level was significantly elevated in the striatal tissues with HAP40 overexpression and decreased when HAP40 was knocked down (Fig. 6A, B). We also examined the total ubiquitin level in the striatum of Cas9 mice injected with AAV-Hap40-gRNA and did not find significant changes (Fig. S9A, B), indicating that such effects are specifically mediated by the presence of mHTT.

Polyubiquitination is an important post-translational mechanism to determine the fate of a protein. The polyubiquitin chain is built on the sequential attachment of ubiquitin monomers through one of the seven lysine residues (K6, K11, K27, K29, K33, K48, and K63) within ubiquitin [49, 50]. Among these lysine residues, the K48-linked polyubiquitin chain is the principal signal for targeting proteins to the 26S proteasome for degradation [51, 52]. We used HEK293 cells stably expressing full-length WT HTT containing 23Q or mHTT containing 120Q. The expression of mHTT in the stable cells is comparable to WT HTT (Fig. S9C). The stable cells were transfected with the Hap40 plasmid, treated with MG132 to block the proteasome activity, and underwent HTT immunoprecipitation to examine its K48 ubiquitination level using a K48-specific antibody. We found that the K48 ubiquitin bands were more visible in mHTT-expressing cells, suggesting that mHTT is likely degraded via K48 ubiquitination (Fig. 6C). Importantly, HAP40 overexpression increased K48 ubiquitination of the precipitated mHTT when compared to the control GFP overexpression (Fig. 6C). On the other hand, knocking down HAP40 via siRNAs reduced K48 ubiquitination of the precipitated mHTT (Fig. S9D, E). Together, our result indicates that HAP40 preferentially binds to mHTT and selective N-terminal mHTT fragments, and promotes K48-linked ubiquitination of mHTT for its degradation in the UPS.

## Discussion

Mounting evidence suggests that HAP40 plays an important role in regulating HTT protein conformation and stability, as the addition of HAP40 is essential for forming a stable HTT complex that can be used to resolve HTT protein structure via cryo-electron microscopy [9]. Moreover, the expression of HAP40 alters the level of HTT in cultured cells [32, 33]. Our current study also shows that the expression of HAP40 and HTT is positively correlated in different brain regions of mice. All the results indicate that HAP40 is closely linked to HTT, both biochemically and functionally. Nonetheless, the potential of the role of HAP40 in HD pathogenesis remains poorly studied. One of the reasons is that HAP40 interacts with the HEAT repeats domain of HTT [9, 53], which is distinct from the exon 1 HTT where the polyQ region is located.

In this study, we found that HAP40 critically regulates mHTT aggregation and toxicity in the mouse brain. Overexpression of HAP40 reduces the formation of mHTT aggregates and ameliorates behavioral deficits caused by mHTT, whereas knockdown of HAP40 enhances mHTT aggregation and aggravates mHTT-induced neurotoxicity. This result is consistent with the previous finding that the knockdown of HAP40 using shRNA promotes the aggregation of mHTT fragments in striatal cells [34]. However, one study reported that deletion of the *Drosophila* homolog of HAP40 (dHAP40) in a fly model expressing human full-length mHTT partially rescued degenerative phenotypes and prolonged lifespan [33]. It is noteworthy that although dHAP40 is a 40 kDa protein that interacts with dHtt, its binding affinity to human HTT is significantly reduced [33]. In addition, the protein sequence homology between dHAP40 and human HAP40 is less than 30%, whereas the homology between mouse HAP40 and human HAP40 is more than 80%. Therefore, the fly model may not fully recapitulate human HAP40 function. Indeed, overexpressing human HAP40 only marginally changed the eye degeneration in the fly model expressing human mHTT [33], which further indicates that it is imperative to study the HTT-HAP40 relationship in a higher organism, such as mice. Among the numerous HD mouse models available, we chose HD140Q KI mice for our study. The main reason is that this mouse model expresses WT and mHTT at endogenous levels so that artifacts caused by HTT overexpression can be avoided. This mouse model has been widely used in the field of HD research [21, 23, 42, 54]. One potential caveat is that this mouse model carries a hyperextended CAG allele with more than 100 repeats. However, given that the intermediate repeat expansion does not result in overt HD-like phenotypes in mice [55], most of the HD KI mouse models currently used contain more than 100 CAG repeats [36, 56–58].

One important finding of our study is that HAP40 preferentially interacts with mHTT when compared with WT HTT. Although the polyQ region does not directly bind to HAP40, the expanded polyQ may alter HTT protein conformation to favor its interaction with HAP40. A similar example is HAP1, another HTT-interacting protein. HAP1 binds to amino acids 171–230 of HTT [59], which does not contain the polyQ region, but HAP1 preferentially binds to mHTT with polyQ expansion [60]. Indeed, in HEK293T cells, the binding affinity of HAP40 to mHTT with 145Q is about one-fold stronger than to HTT with 23Q [33], which supports our conclusion. It should be noted that efforts have been devoted to studying the structure of HTT in the context of different polyQ lengths, but the results remain inconsistent: there are two studies indicating that the polyQ expansion does not alter the HTT-HAP40 structure [53, 61], whereas two other studies presented conflicting results showing significant differences in the structures of WT and mHTT protein [10, 62]. It is technically challenging to purify mHTT protein for structural studies, which typically rely on transfected proteins from cultured cells. Protein overexpression could trigger the unfolded protein response, which potentially affects the HTT structure. In addition, post-translational modifications of HTT in the neurons of the brain may influence HTT-HAP40 interaction, which may not be revealed by the in vitro structural study. It is also important to determine the polyQ threshold for the preferential binding of mHTT to HAP40 in the brain. However, as most HD KI mouse models express mHTT with more than 100 polyQ repeats, postmortem brain tissues from HD patients carrying various polyQ lengths are needed to elucidate this point.

It has been suggested that HAP40 is involved in either UPS or autophagy activities in cell models [34, 35]. Our result based on mouse brain demonstrated that manipulation of HAP40 expression changes overall ubiquitination level, but not proteins related to endosome or autophagy. A previous study also reported co-staining of endogenous HAP40 and ubiquitin in a distinct structure named intranuclear rodlet (INR) in the mouse brain [63]. Collectively, these results favor the idea that HAP40 is more closely linked to the UPS, though the exact mechanism remains to be investigated. In addition, we found that HAP40 promotes K48-linked ubiquitination of mHTT. K48-linked ubiquitination is a well-established signal that leads to protein degradation [51] and has been shown to enhance mHTT degradation through the UPS [64]. Therefore, HAP40 can promote the clearance of soluble mHTT, which leads to decreased mHTT aggregation.

Our study identified HAP40 as a pivotal regulator of mHTT toxicity, as the reduction of HAP40 in the striatum of HD140Q KI mice led to severe neuronal loss and motor deficits, and overexpression of HAP40 improved the behavioral performances of HD140Q KI mice. The modest behavioral improvement could be due to the limited infection area mediated by AAV injection. Future studies using genetic approaches to knockout or overexpress HAP40 are warranted to further validate the role of HAP40 in HD pathogenesis. Interestingly, in our previous publication, we found a similar outcome in HD140Q KI mice when manipulating the expression of HAP1 [41]. As the phenotypes of HD140Q KI mice are relatively mild, with overt neuronal loss only at 20–26 months of age [36, 42], the rapid neurodegeneration induced by HAP40 or HAP1 reduction indicates that mHTT toxicity is strongly influenced by certain interacting partners. On the other hand, HTT-lowering strategies are being actively pursued as potential therapies for HD [65, 66], but non-allele selective reduction of HTT could cause safety issues [67, 68]. Identification of HAP40 as a candidate that specifically modulates mHTT toxicity offers a new potential target to treat HD.

## Methods

### Antibodies and reagents

The antibodies were purchased from commercial companies or produced by our laboratory previously and listed as follows: HAP40 (Sigma, HPA046960), HTT (Millipore, MAB2166; abcam, ab109115; Cell signaling, 5656S), mEM48 (self-made), 1C2 (Millipore, MAB1574), HA tag (Cell signaling, 3724S), Vinculin (Millipore, MAB3574), NeuN (abcam, ab177487), GFAP (abcam, ab7260), total ubiquitin (Cell signaling, 3936S), K48 ubiquitin (Cell signaling, 8081S), Cas9 (Millipore, MAC133), EEA1 (abcam, ab2900), Rab5a (Cell signaling, 46449T), LC3 (Cell signaling, 2775S), DARPP32 (Santa Cruz, sc-271111), β3-tubulin (Sigma, T8328), F4/80 (Novus, NB600-404). The N-terminal HTT plasmids were previously generated in our laboratory [64].

The sequences of siRNAs are listed below: Ctrl-siRNA, sense, 5′-UUC UCC GAA CGU GUC ACG UTT-3′, antisense, 5′-ACG TGA CAC GTT CGG AGA ATT-3′; siHap40_613, Sense: 5′-GGC GCU AUU UAC ACG CAU GTT-3′, antisense: 5′-CAU GCG UGU AAA UAG CGC CTT-3′; siHap40_986, sense, 5′-GAG GAG CUG UUU CUG UUA UTT-3′, antisense, 5′-AUA ACA GAA ACA GCU CCU CTT-3′; siHAP40_959, sense, 5′-GCU UCC CGA GGA GCU CUU UCU TT-3′, antisense, 5′-AGA AAG AGC UCC UCG GGA AGC TT-3′; siHAP40_996, sense, 5′-GUC AUG GCU ACC CAC GAA ATT-3′, antisense: 5′-UUU CGU CCC UAG CCA UGA CTT-3′; siHAP40_1073, sense, 5′-GCA GAA CCA CCU CCU UCA CCU TT-3′, antisense: 5′-AGG UGA AGG AGG UGG UUC UGC TT-3′. The siRNAs were purchased from GenePharma.

### Animals

Wild-type C57BL6J mice were purchased from Guangdong Medical Laboratory Animal Center (Guangzhou, China). HD140Q KI mice were gifts from Dr. Zhong Pei at Sun Yat-sen University. The polyQ length of mHTT in these mice is around 160 repeats [69]. Cre-dependent Cas9 transgenic mice were purchased from The Jackson Laboratory (Stock No: 024857, Rosa26-LSL-Cas9 KI) and crossed with EIIA-Cre transgenic mice to generate germline transmissible mice that ubiquitously express Cas9 in all tissues. HD140Q/Cas9 mice were generated by crossing HD140Q KI mice with germline Cas9 mice. All the mice were maintained in a 12-h light/dark cycle in the Division of Animal Resources of Jinan University. Both male and female mice were used in every experiment of this study.

For genotyping, the genomic DNA was extracted using Mouse Tail Genomic DNA kit (CWBIO, CW2094S), and the following primers were used for PCR amplification: HD140Q KI, HD-forward (5′-ACT GCT AAG TGG CGC CGC GTA G-3′), WT-forward (5′-GCG GCT GAG GGG GTT GA-3′), and common reverse (5′-GAG GCA GCA GCG GCT GTG CCT G-3′); Cas9, forward (5′- TCG AAA ATC TGT GGG AAG TC-3′) and reverse (5′- AAG GGA GCT GCA GTG GAG TA-3′).

### Cell culture

HEK293 and N2A cells were purchased from ATCC and cultured in DMEM (C11995500CP, Gibco, USA) containing 10% (v/v) fetal bovine serum (FBS) (04-001-1B, Biological Industries, Germany), 100 U/ml penicillin and streptomycin (15140163, Gibco, USA). HEK293 cell lines stably expressing full-length HTT with 23Q (23Q-HEK293) or 120Q (120Q-HEK293) were established previously in our laboratory and selected by 500 μg/ml hygromycin B (Invivogen, ant-hg-5) in culture medium. For transient transfection of plasmids or siRNAs, lipofectamine 3000 (L3000001, Invitrogen, USA) was used according to the manufacturer’s protocol, and the cells were harvested 48 h after transfection. For proteasomal inhibition, 48 h after transfection, MG132 was added to the culture medium for a final concentration of 10 µM. The cells were harvested 12 h after MG132 treatment.

### Adeno-associated virus (AAV) construction

Mouse *Hap40* cDNA with C-terminal HA tag was generated by PCR using the following primers: forward (5′- TAG GAT CCG CCA CCA TGG CTG CGG GCT CTG C-3′), reverse (5′- TCA TGA TAT CTC AAG CGT AAT CTG GAA CAT CGT ATG GC-3′) and cloned into the AAV plasmid under the control of ubiquiting C (UBC) promoter. PX551 (AAV-pMecp2-Cas9) and PX552 were gifts from Dr. Feng Zhang at the Massachusetts Institute of Technology (Addgene plasmid #60957, 60958). *Hap40* gRNA sequences used include #2, GAG CCC GCA GCC ATG TTG ACC GG; #50, CCT CCT TGG GCG GCG GTG CCT GG; #93, CTT CTT GGC ACG CTA TCG GCA GG; control, ACC GGA AGA GCG ACC TCT TCT. These sequences were cloned into PX552 vector. All the plasmids were packaged into AAV9 virus by PackGene Biotech Inc.

### Stereotaxic surgery

The method for stereotaxic surgery was the same as described previously [21]. First, the mice were anesthetized with continuous 1.5% isoflurane inhalation and fixed on a stereotaxic instrument (RWD, 69100). The hair around the surgical site was removed using a disinfected scissor and the skin was sterilized with 70% alcohol. Then, the skin was cut to expose the bregma of the skull, and the injection sites were identified according to the distance from the bregma. Microdrill was used to make small holes in the skull, and the AAV (1E + 13 GC/ml) was injected into the brain using 33G microsyringe (Hamilton. NanoFil-10ul-1) at the speed of 200 nL/min. The volume of AAV was 1 μl for each injection site. The microsyringe was left in the injection site for 10 min, and then slowly withdrawn. After suturing the wound, the mice were placed on a heated blanket until awakened. The injection coordinates used were as follows: striatum, anterior-posterior = +0.55 mm, medial-lateral = ±2 mm, dorsal-ventral =−3.5 mm; cortex, anterior-posterior = +0.55 mm, medial-lateral = ±2 mm, dorsal-ventral =−1.5 mm. All post-surgical mice were closely monitored.

### Western blot

Cell samples were washed with PBS 3 times and collected; brain tissues were ground 20–30 times using Dounce Tissue Grinders (Thermo Fisher, K8853000002). Then the samples were lysed in RIPA buffer (50 mM Tris, pH 8.0, 150 mM NaCl, 1 mM EDTA pH 8.0, 1 mM EGTA pH 8.0, 0.1% SDS, 0.5% DOC, and 1% Triton X-100) with Protease inhibitor cocktail (Mei5bio, MF182-plus-10), and sonicated 6 times. The protein in the samples was separated by SDS-PAGE electrophoresis using SurePAGE™ Bis-Tris Gels (GenScript, M00652), and transferred onto a nitrocellulose membrane. The membranes were blocked with 5% skim milk at room temperature for 1 h and incubated with the corresponding primary antibodies in 3% BSA at 4 °C overnight. The next day, the membranes were washed three times with 1× PBS and incubated with HRP-conjugated secondary antibodies in 5% skim milk at room temperature for 1 h. The signals were developed with ECL solution (Millipore, WBKLS0500) after washing three times in 1 X PBS. The images were acquired digitally using Clinx ChemiScope 6300.

### Immunohistochemistry

The mice were anesthetized and intracardially perfused with warm 0.9% saline solution, followed by 4% ice paraformaldehyde (PFA) in 0.1 M PB solution. The brains of the mice were separated and fixed overnight in 4% PFA solution and transferred to 15% sucrose for 24 h, and 30% sucrose for another 24 h. The brains were embedded in the OCT solution (Sakura, 4583), sectioned at 30 μm in a cryostat (Thermo Fisher), and soaked in 0.1 M PB buffer containing 0.03% NaN_3_ for storage. Brain slices were blocked in blocking buffer (3% BSA/2% goat serum/0.1% Triton X-100/1 X PBS) for 1 h and incubated with primary antibodies in blocking buffer at 4 °C overnight. After washing with 1× PBS three times, the brain slices were incubated with secondary antibodies for 10 min and developed with the Mouse and Rabbit Specific HRP/DAB (ABC) Detection IHC kit (Abcam, ab64264) following the manufacturer’s protocol.

### Immunofluorescent staining

The same method described in Immunohistochemistry was used to fix and prepare brain slices for immunofluorescent staining. The brain slices were penetrated with 0.5% Triton X-100 in 1× PBS for 30 min and blocked with a blocking buffer for 1 h at room temperature. The brain slices were incubated with primary antibodies at 4 °C overnight. The next day, the slices were washed with 1× PBS three times and incubated with secondary fluorescent antibodies for 1 h and DAPI for 10 min. The slices were observed and captured using a Zeiss AX10 Axio Microscope or Olympus FV3000 confocal laser scanning microscope. To quantify mHTT aggregates, the images were opened in the ImageJ software (Version 1.53a). The aggregates were manually identified and automatically counted using the “multi-point” function of ImageJ. The counting was performed by personnel who were blind to the experimental groups.

### Immunoprecipitation

The cell or brain samples were collected and lysed in 1% NP-40 buffer (150 mM NaCl, 1% NP-40, 2 mM EDTA, 50 mM Tris, pH 8.0, 1 mM PMSF) with protease inhibitor cocktail. After 1 h of lysis on ice, the samples were centrifuged at 14,000 × *g* for 20 min and the supernatant was collected. After pre-clearing with 50 μL of Dynabeads™ Protein G beads (Thermo Fisher, 10004D), 500 μg of protein was incubated with the primary antibodies at 4 °C overnight. The next day, the samples were incubated with 50 μL of Protein G beads at 4 °C for 1 h. The beads were collected by the magnetic frame and boiled with 1 X SDS loading buffer at 95 °C for 10 min.

### Behavioral tests

The motor ability of the mice was accessed by the rotarod test, balance beam test, and grip strength test. The mice tested were littermates randomly assigned to the experimental groups. Each group contained age and sex-matched mice. The tests were performed by personnel who were blind to the experimental groups. No animals were excluded from the analysis. For the rotarod test, the mice were trained on the rotarod at 5 RPM for 5 min, three times daily for three consecutive days. In the actual test, the speed of the rotarod was set to slowly accelerate to 40 RPM over a period of 5 min. For the balance beam test, the mice were placed on a 100 cm-long, 6 mm-wide beam hanging 50 cm above the floor. The mice were trained to move from one end of the beam to the other for three consecutive days. In the actual test, the time that the mice moved through 80 cm on the beam was recorded. For the grip strength test, the mice were measured by a grip strength meter three times. All three tests were performed twice per month.

### Statistical analysis

The data were analyzed by the Prism 8 (GraphPad) software. For comparisons between two groups, a two-tailed Student’s *t*-test was used. For three or more groups, one-way ANOVA with Tukey’s multiple comparisons tests was used. For the longitudinal behavioral tests, two-way ANOVA with Sidak post-tests was used. The quantitative results were presented as mean ± SEM. *P* < 0.05 was considered a significant difference.

### Supplementary information

Fig. S1-S9

Full and uncropped western blots

## Data Availability

All datasets generated and analyzed during this study are included in this article.

## Materials availability

Research materials are available from the corresponding authors (xjli33@jnu.edu.cn or syang33@jnu.edu.cn) on reasonable request.
