# Supplementary material for: HAP40 modulates mutant Huntingtin aggregation and toxicity in Huntington’s disease mice
Source: Cell Death Dis. 2024 May 14;15(5):337. doi: 10.1038/s41419-024-06716-4 (PMC11094052; doi:10.1038/s41419-024-06716-4)
Supplement: Supplementary file 1 — Fig. S1-S9 [file 41419_2024_6716_MOESM1_ESM.pdf]

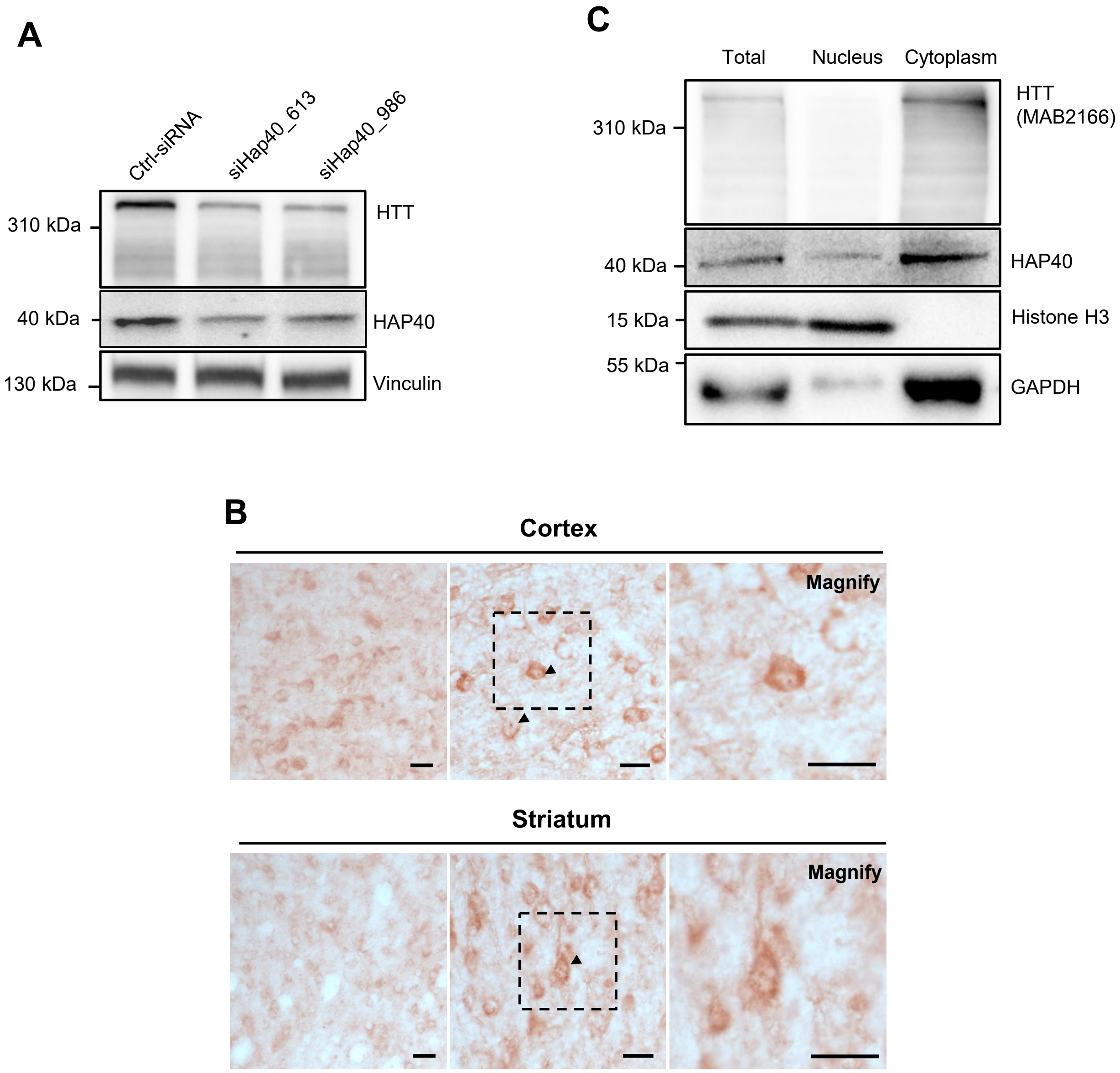

Figure S1. HAP40 is mainly localized in the cytoplasm. (A) N2A cells were transfected with two siRNAs targeting *Hap40* (siHap40_613 and siHap40_986) or scramble siRNA (Ctrl-siRNA). Western blotting results confirmed the reduction of HAP40 protein in cells transfected with siHap40_613 or siHap40_986. HTT protein expression was also decreased. **(B)** Immunohistochemistry of the cortex and striatum slices showed that HAP40 is predominately localized in the cytoplasm, and also displays punctate staining in the nucleus (indicated by black arrowheads; 40X, scale bar: 20 μm). **(C)** Subcellular fractionation of brain tissues was performed to separate nucleus and cytoplasm. Both HAP40 and HTT were predominantly found in the cytoplasm. Histone H3 was used as a nuclear marker, and GAPDH was used as a cytoplasmic marker.

Fig. S2

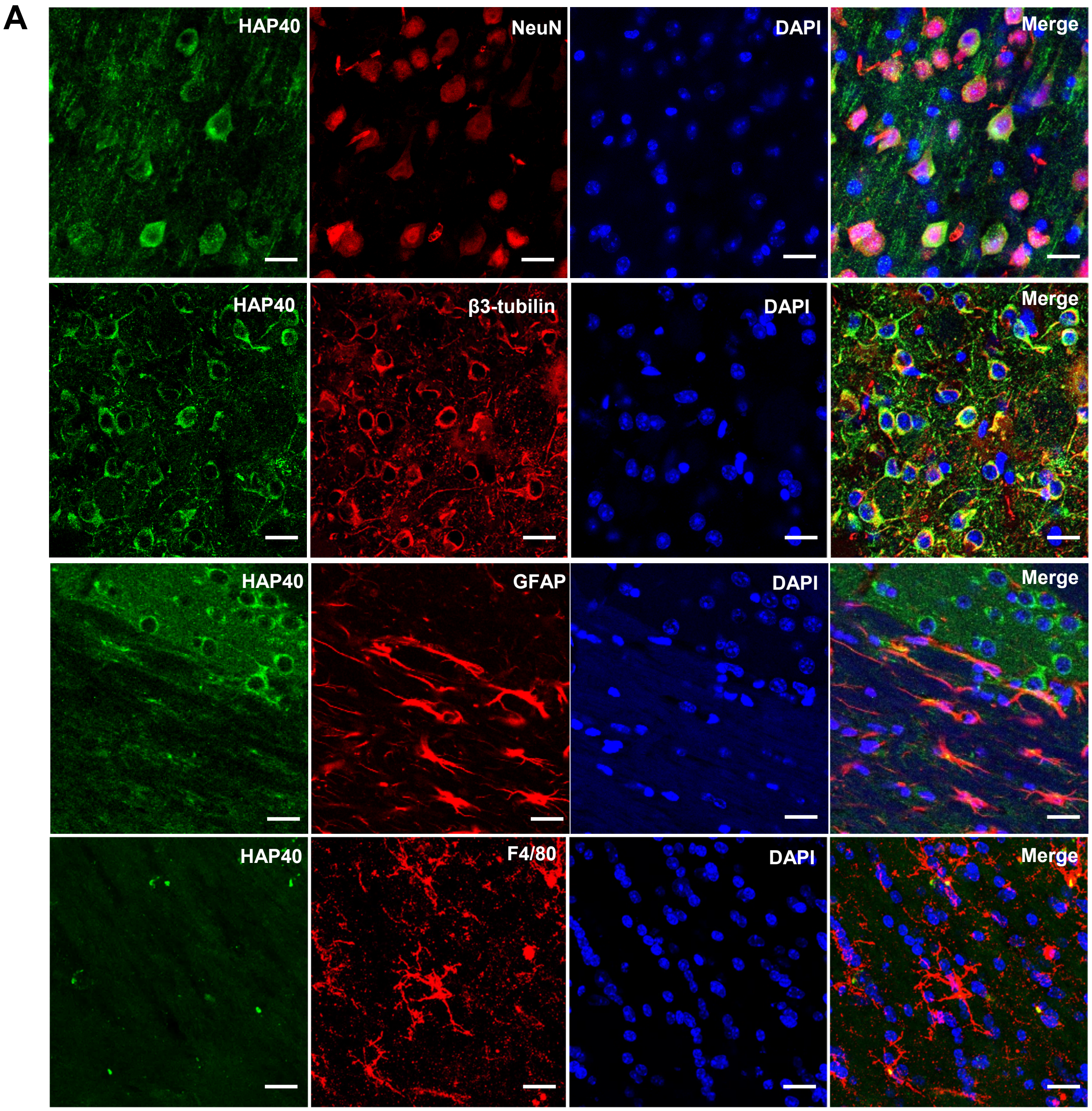

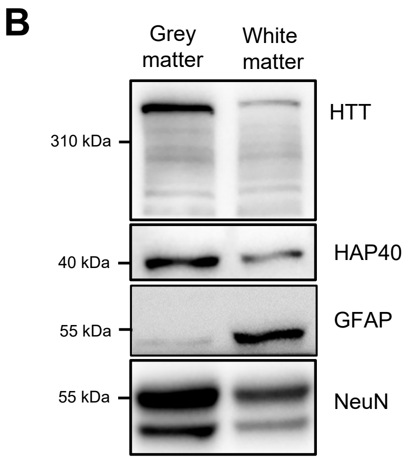

Figure S2. HAP40 is mainly expressed in neurons. (A) Double immunofluorescent staining was performed using cortical slices of WT mice. HAP40 is mainly present in NeuN and β3-tubilin-positive cells, but not in GFAP or F4/80-positive cells (40X, scale bar: 20 μm). **(B)** Western blotting results show that more HAP40 and HTT are expressed in the grey matter compared the white matter. NeuN was used as a neuronal marker, and GFAP was used as an astrocyte marker.

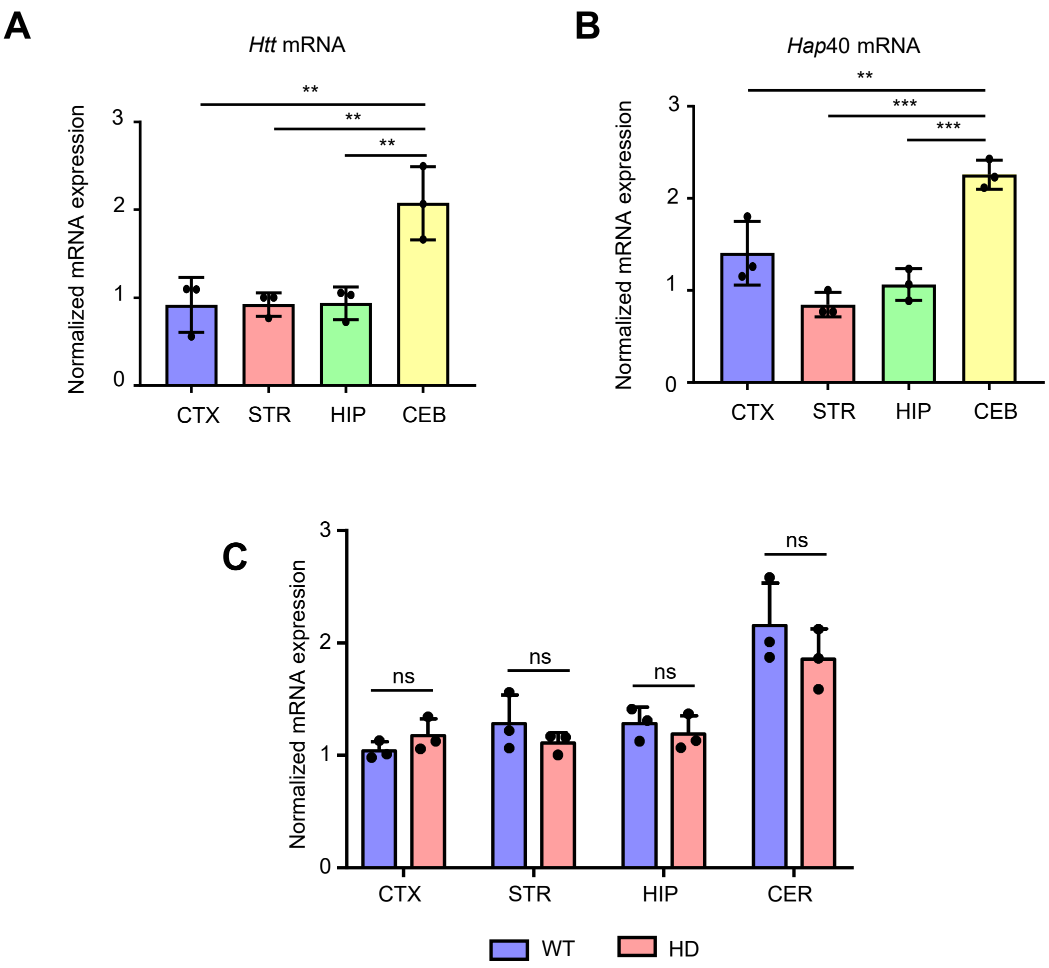

Figure S3. Characterization of *Htt* and *Hap40* mRNA levels. (A) The level of *Htt* mRNA in different brain regions of WT mice. **(B)** The level of *Hap40* mRNA in different brain regions of WT mice (n = 3, one-way ANOVA with Tukey post-tests; CTX vs CEB, *P* = 0.0020; STR vs CEB, *P* = 0.0024; HIP vs CEB, *P* = 0.0023). **(C)** The levels of *Hap40* mRNA in 10-month-old WT and HD140Q KI mice (n = 3, two-tailed student t test, CTX, *P* = 0.1070; STR, *P* = 0.3520; HIP, *P* = 0.1714; CEB, *P* = 0.3406). CTX, cortex; STR, striatum; HIP, hippocampus; CEB, cerebellum. Ns, not significant; **, *P* < 0.01; ***, *P* < 0.001. Data are presented as mean values ± SEM.

A

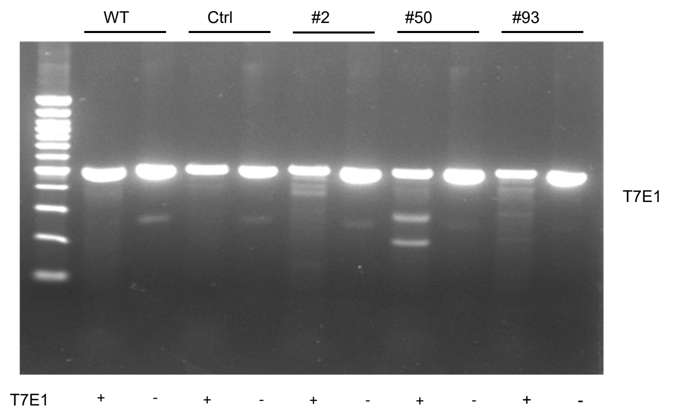

B

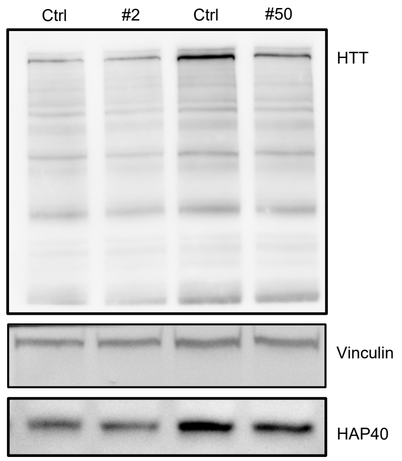

Figure S4. Verification of HAP40 knockdown by CRISPR/Cas9. (A) T7E1 assay was performed to test the targeting efficiency of different Hap40-gRNAs (#2, #50 and #93) in N2A cells. **(B)** Hap40-gRNA/Cas9 viruses were injected into the striatum of WT mice. The expression of HAP40 was examined one month after injection.

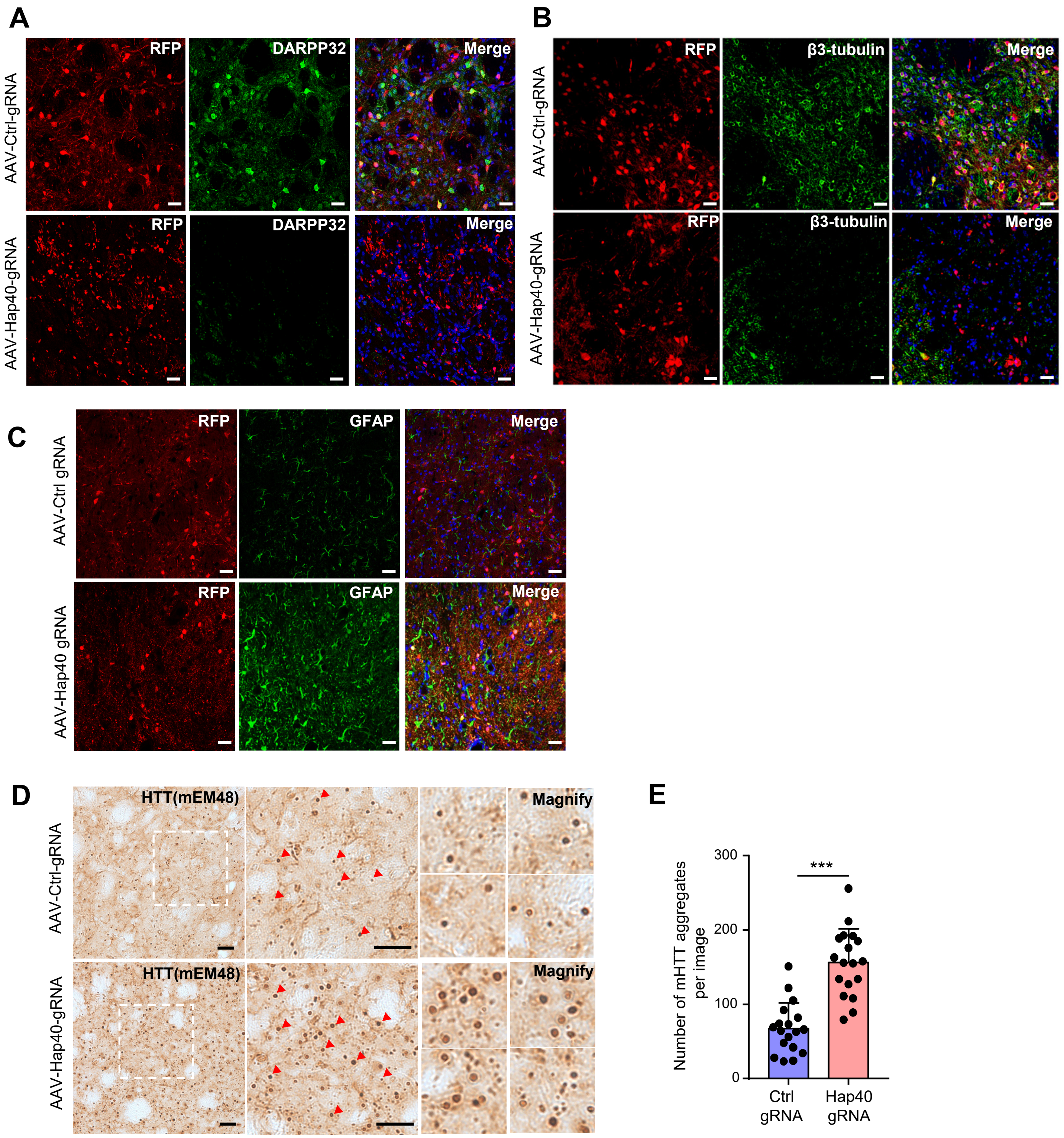

Figure S5. Further analysis of HAP40 knockdown in the HD140Q KI mice. (A) Immunofluorescent staining of DARPP32 in the striatum of HD140Q KI mice injected with either AAV-Ctrl-gRNA or AAV-Hap40-gRNA (40X, scale bar: 20 μm). **(B)** Immunofluorescent staining of β3-tubulin in the striatum of HD140Q KI mice injected with either AAV-Ctrl-gRNA or AAV-Hap40-gRNA (40X, scale bar: 20 μm). **(C)** Immunofluorescent staining of GFAP in the striatum of HD140Q KI mice injected with either AAV-Ctrl-gRNA or AAV-Hap40-gRNA (40X, scale bar: 20 μm). **(D)** Immunohistochemical staining showed that the number of mHTT aggregates were increased in the AAV-Hap40-gRNA injected striatum, compared to the AAV-Ctrl-gRNA injected striatum (red arrowheads indicate mHTT aggregates (40X, scale bar: 20 μm). **(E)** Quantitative results of the number of mHTT aggregates in the immunohistochemical staining images (n = 18 from three mice, two-tailed student t test, $P < 0.0001$). ***, $P < 0.001$. Data are presented as mean values ± SEM.

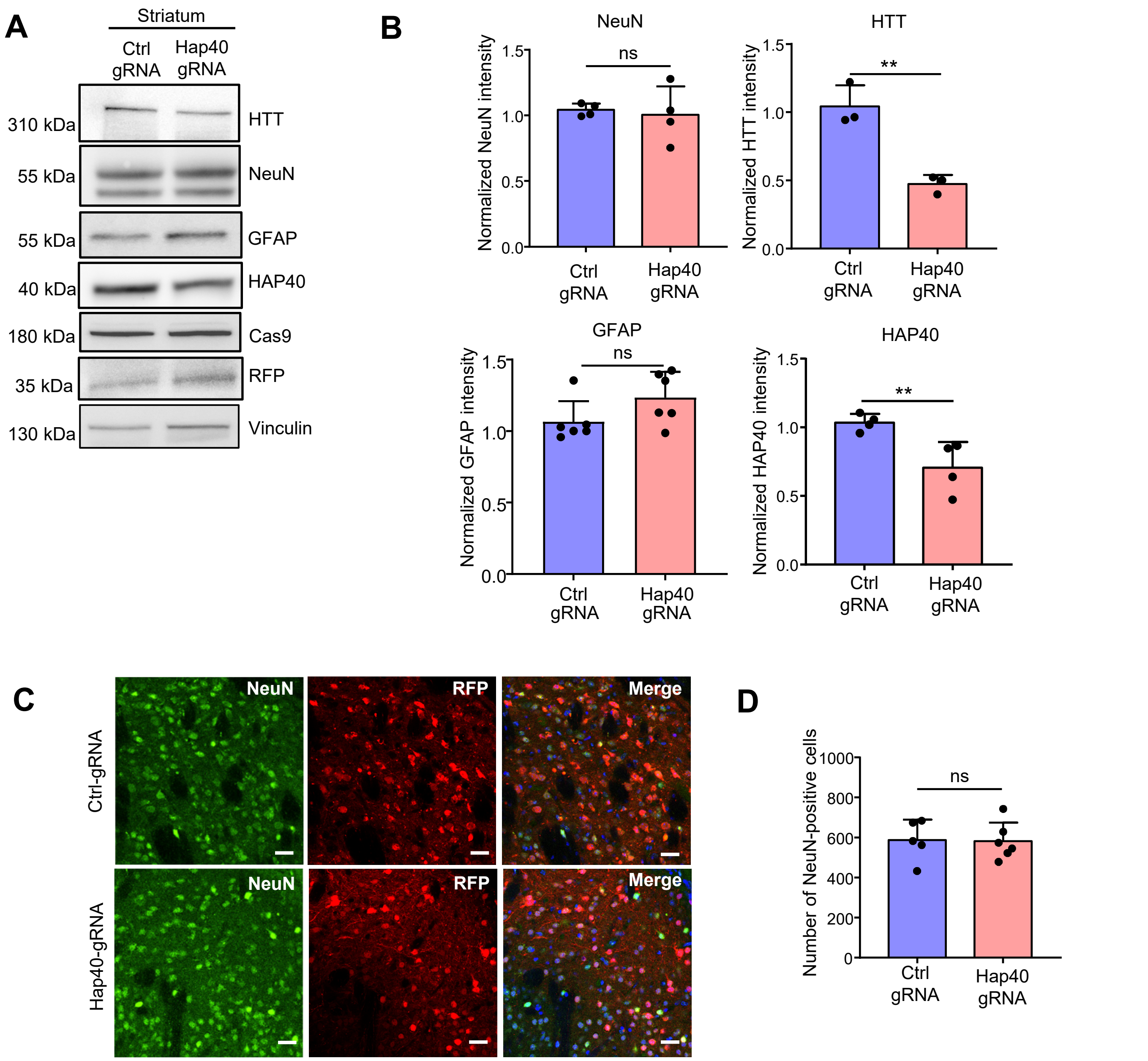

Figure S6 HAP40 reduction does not cause neurodegeneration in the striatum of germline Cas9 mice. (A) Western blotting analysis of HTT, NeuN, GFAP, and HAP40 expression in the striatum of Cas9 mice injected with either AAV-ctrl-gRNA or AAV-Hap40-gRNA. Vinculin was used as a loading control. **(B)** Quantitative analysis of the results in Fig. S6A (n = 3 - 6, two-tailed student t test; NeuN, *P* = 0.8183; HTT, *P* = 0.0034; GFAP, *P* = 0.2142; HAP40, *P* = 0.0015). **(C)** Immunofluorescent staining of NeuN in the striatal slices of Cas9 mice injected with AAV-ctrl-gRNA or AAV-Hap40-gRNA (40X, scale bar: 20 μm). **(D)** Quantitative analysis of the number of NeuN-positive cells in the immunofluorescent images (n = 6, two-tailed student t test, *P* = 0.8473). Ns, non-significant; **, $P < 0.01$. Data are presented as mean values ± SEM.

Fig. S7

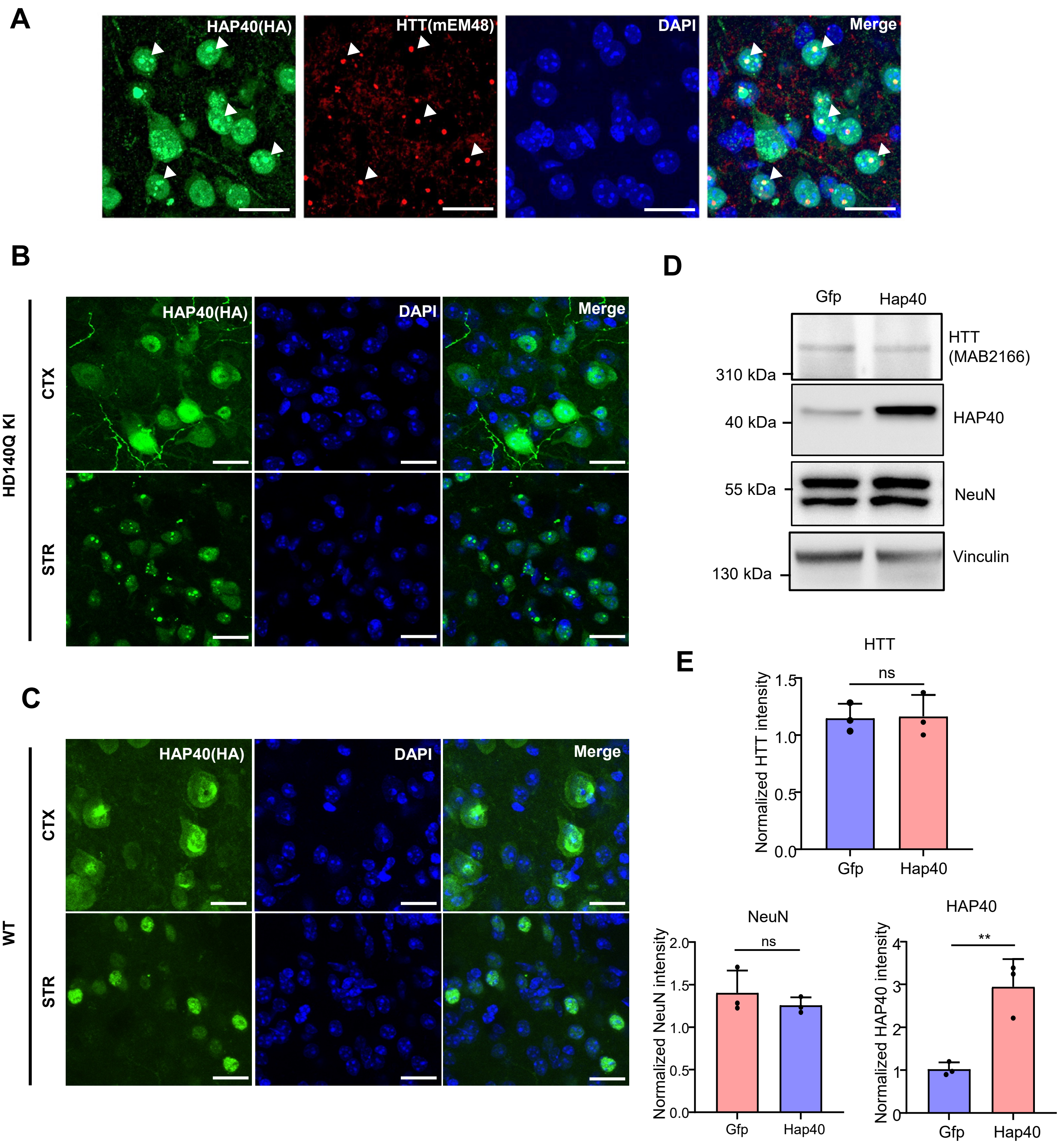

Figure S7. The exogenous HAP40 co-localizes with nuclear mHTT aggregates in the striatum of HD140Q KI mice. (A) Double immunostaining showed that the exogenously expressed HAP40 (detected by HA antibody) colocalizes with nuclear mHTT aggregates (detected by mEM48 antibody) in the striatum of HD140Q KI mice (white arrowheads indicate mHTT aggregates in the nucleus; 40X, scale bar: 20 μm). **(B)** In HD140Q KI mice, the exogenously expressed HAP40 (detected by HA antibody) is diffused in the cortex but shows punctate patterns in the nucleus of striatal cells. **(C)** In WT mice, the exogenously expressed HAP40 is diffused both in the cortex and striatum (CTX, cortex; STR, striatum; 40X, scale bar: 20 μm). **(D)** Western blotting analysis of HTT, HAP40 and NeuN protein expression in the striatum of WT mice injected with AAV-Gfp or AAV-Hap40. Vinculin was used as a loading control. **(E)** Quantitative analysis of HTT, HAP40, and NeuN protein expression (n = 3, two-tailed student t test; HTT, *P* = 0.8557; HAP40, *P* = 0.0072; NeuN, *P* = 0.5322). Ns, non-significant, **, *P* < 0.01. Data are presented as mean values ± SEM.

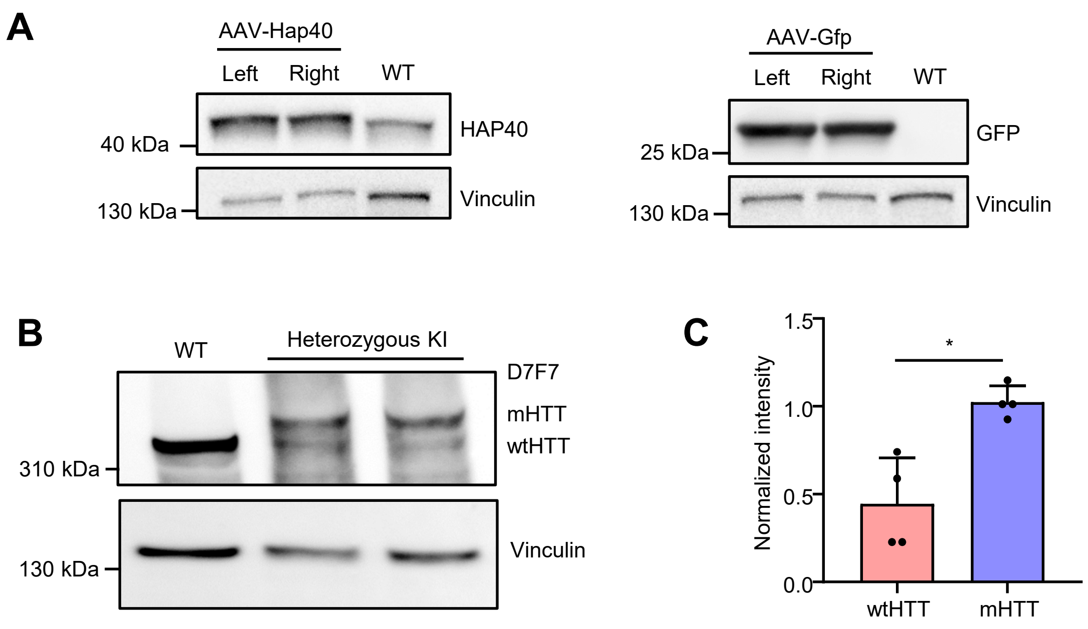

Figure S8. The expression of AAV-Hap40 persists seven months after viral injection in the striatum. (A) Western blotting result showing the expression of HAP40 and GFP in both sides of the striatum in 13-month-old HD140Q KI mice injected with either AAV-Hap40 or AAV-Gfp. Vinculin was used as a loading control. **(B)** Western blotting result of HTT expression in the brain of WT and heterozygous HD140Q KI mice using the D7F7 antibody. **(C)** Quantitative analysis of wtHTT and mHTT in the brain of heterozygous HD140Q KI mice indicates that D7F7 preferentially reacts with mHTT (n = 4, two-tailed student t test, P = 0.0278). *, P < 0.05. Data are presented as mean values ± SEM.

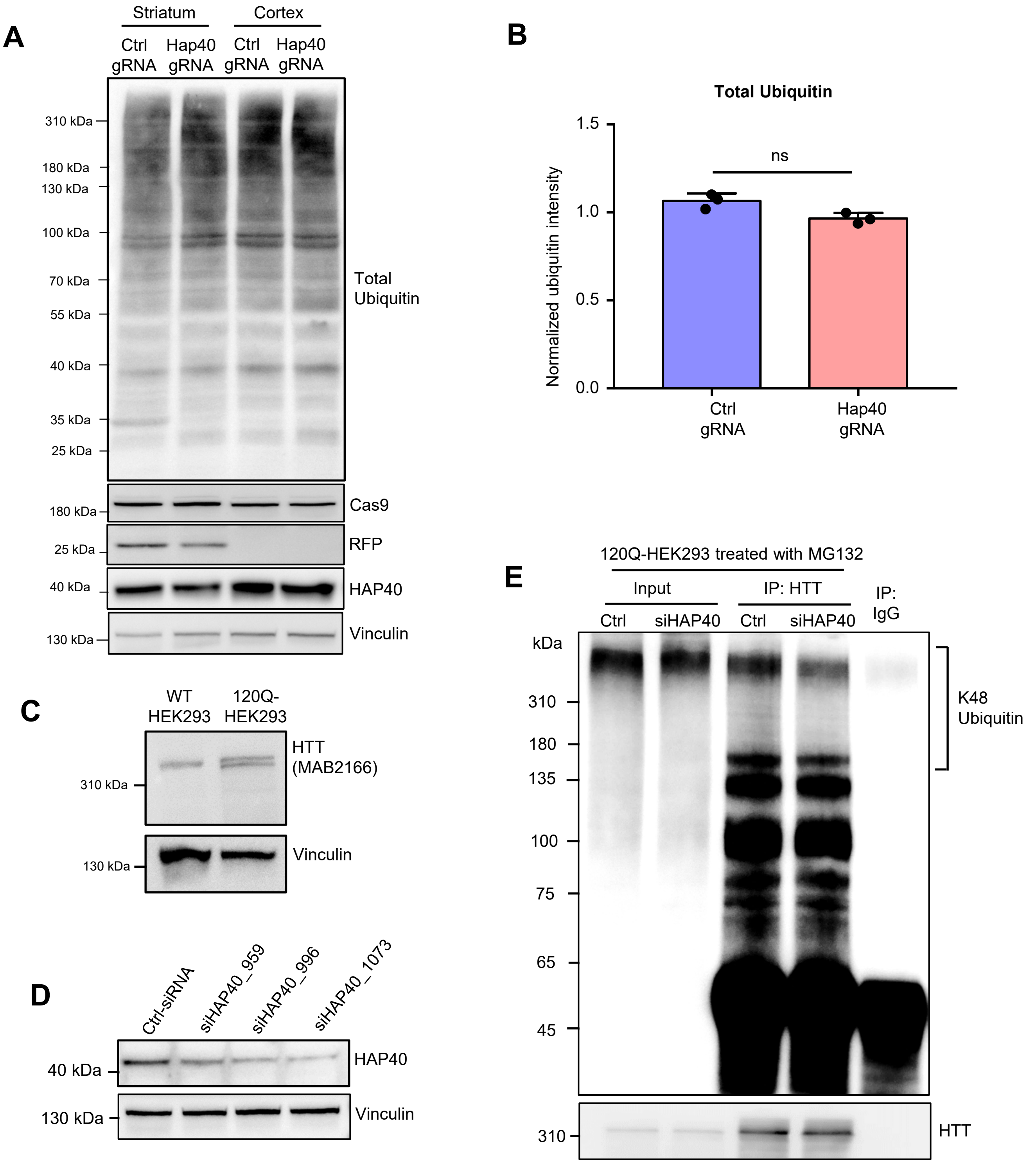

Figure S9. HAP40 reduction does not alter ubiquitination level in WT mice. (A) Western blotting analysis of the total ubiquitination level in the striatum of Cas9 mice injected with AAV-Hap40-gRNA or AAV-Ctrl-gRNA. **(B)** Quantitative analysis of the total ubiquitin level in the striatum of the virus-injected mice (n = 3, two-tailed student t test, P = 0.09664). **(C)** Western blotting analysis of HTT expression in WT and 120Q-HEK293 stable cells. **(D)** HEK293 cells were transfected with three siRNAs targeting *HAP40* (siHAP40_959, siHAP40_996 and siHAP40_1073) or scramble siRNA (Ctrl-siRNA). Western blotting results confirmed the reduction of HAP40 in cells transfected with HAP40 siRNAs. **(E)** Co-immunoprecipitation assay was performed using 120Q-HEK293 cells transfected with the *HAP40* siRNA for 48 hours, and then treated with MG132 for another 12 hours. HTT was precipitated by the EPR5526 antibody and K48 ubiquitin antibody was used to detect K48-linked ubiquitination. Reduced K48-ubiquitinated bands in the siHAP40-treated sample are indicated by the bracket. Ns, non-significant. Data are presented as mean values $\pm$ SEM.
