# Supplementary material for: HAP40 modulates mutant Huntingtin aggregation and toxicity in Huntington’s disease mice
Source: Cell Death Dis. 2024 May 14;15(5):337. doi: 10.1038/s41419-024-06716-4 (PMC11094052; doi:10.1038/s41419-024-06716-4)

Fig. 1B, wtHTT(MAB2166)

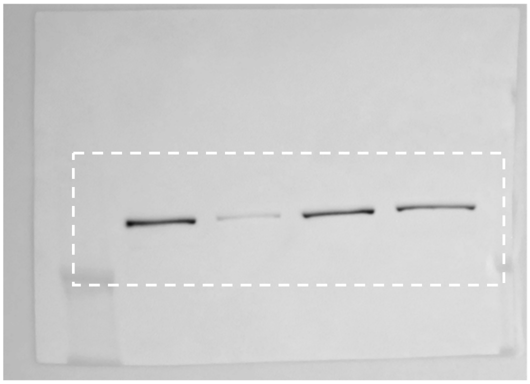

Fig. 1B, wtHTT (D7F7)

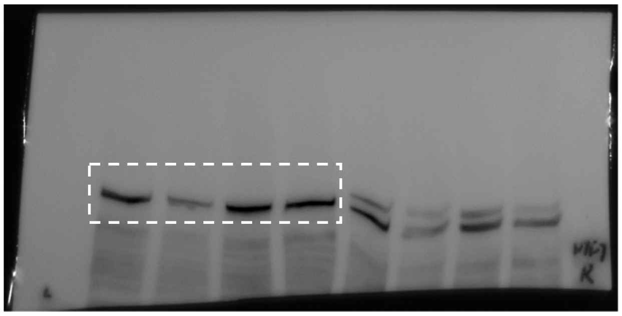

Fig. 1B, HAP40

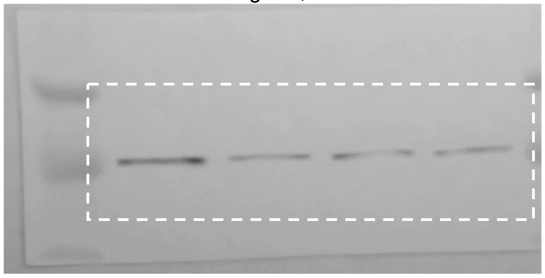

Fig. 1B, Vinculin

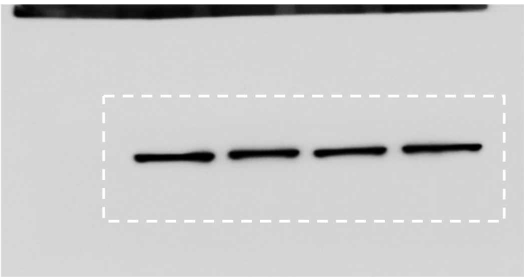

Fig. 1D, Aggregated mHTT

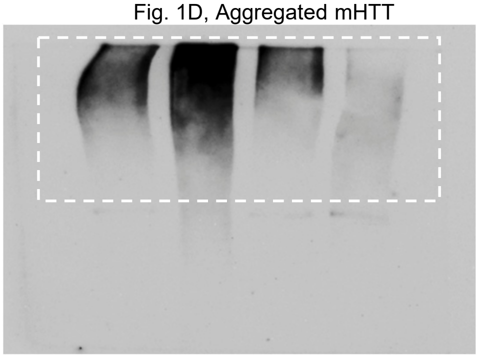

Fig. 1D, mHTT (D7F7)

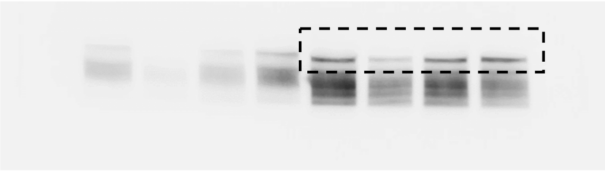

Fig. 1D, mHTT (1C2)

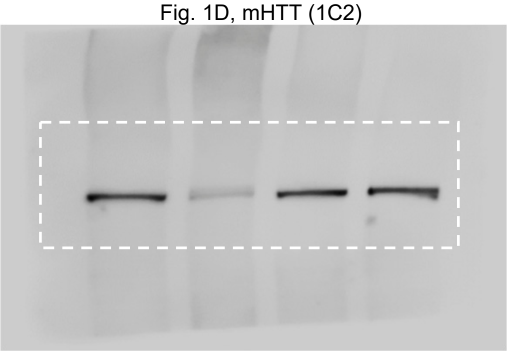

Fig. 1D, HAP40

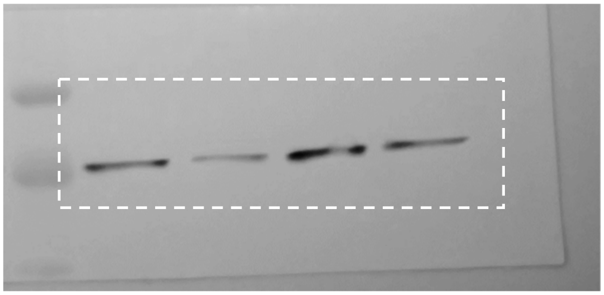

Fig. 1D, Vinculin

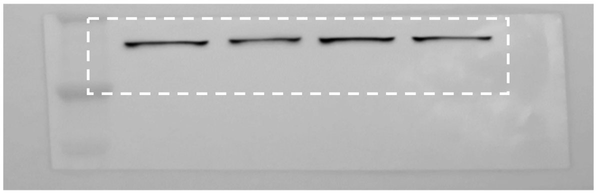

Fig. 1G, HTT

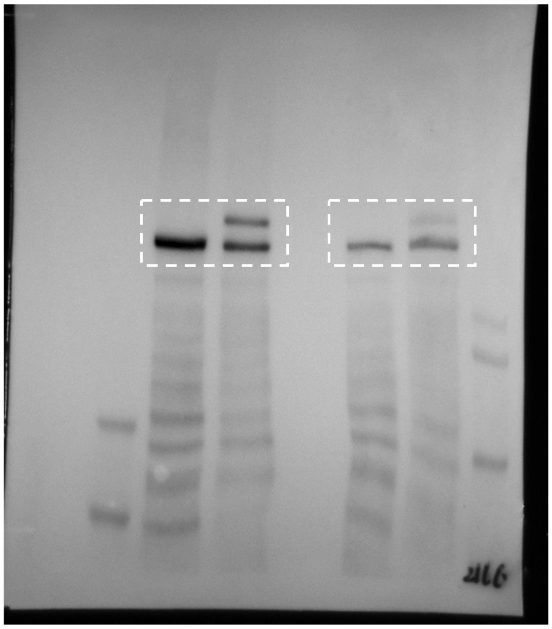

Fig. 1G, HAP40

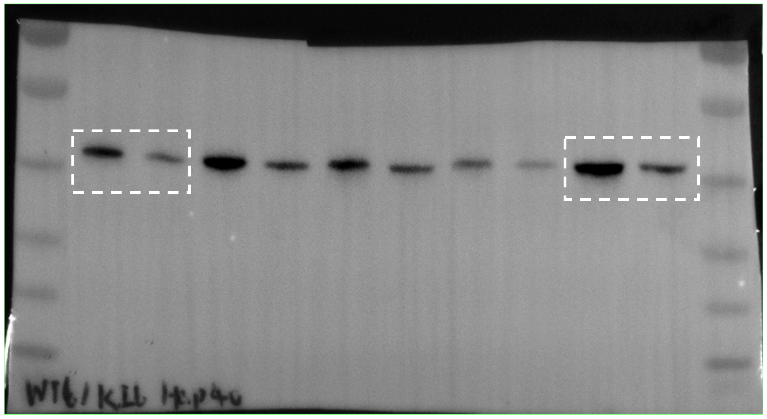

Fig. 1G, Vinculin

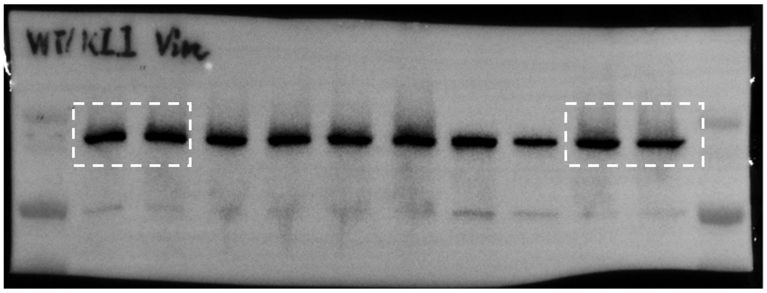

Uncropped images in Figure 2

Fig. 2B, mHTT

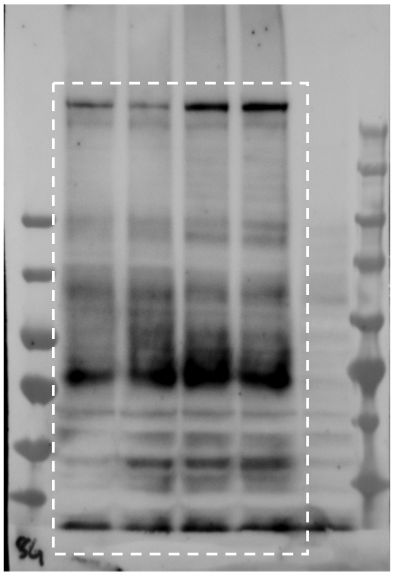

Fig. 2B, Aggregated mHTT

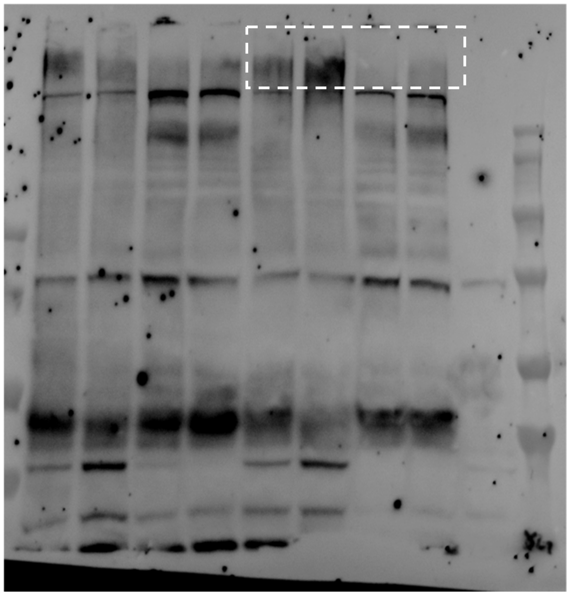

Fig. 2B, RFP

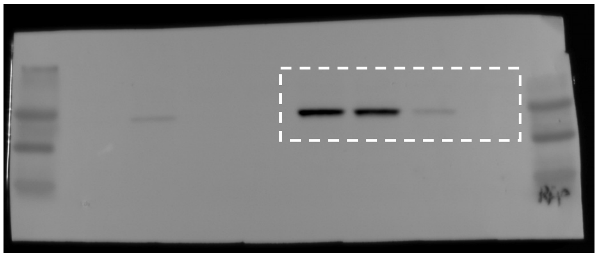

Fig. 2B, Cas9

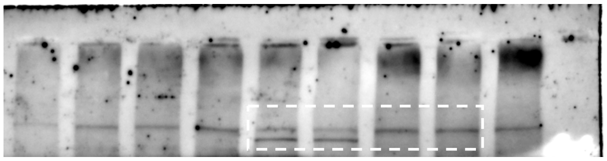

Fig. 2B, Vinculin

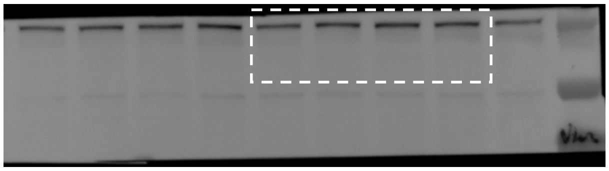

Fig. 2B, HAP40

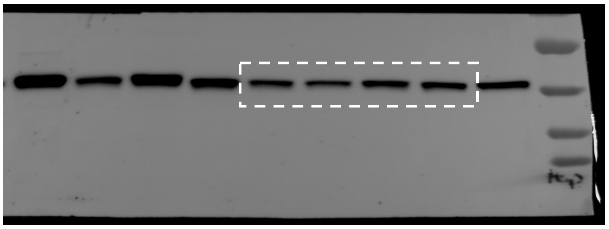

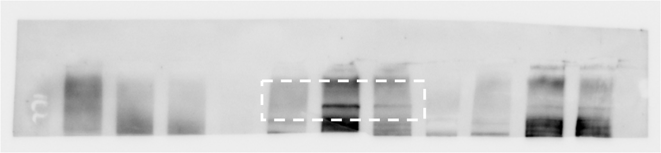

Fig. 3E, mHTT

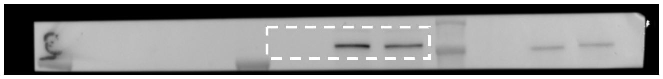

Fig. 3E, Cas9

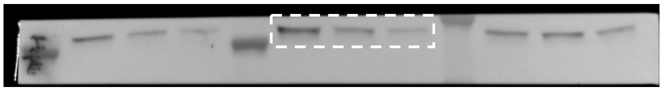

Fig. 3E, HAP40

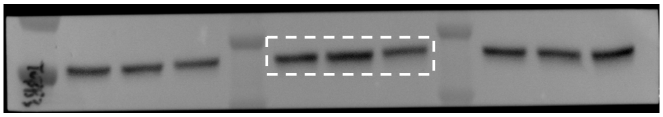

Fig. 3E, β3-tubulin

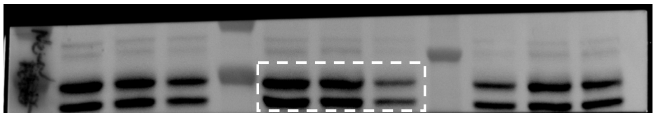

Fig.3E, NeuN

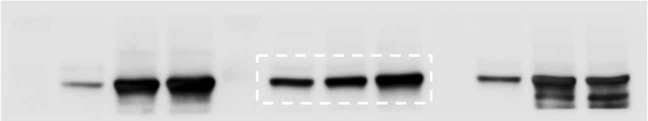

Fig. 3E, GFAP

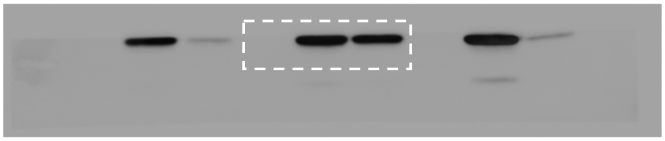

Fig. 3E, RFP

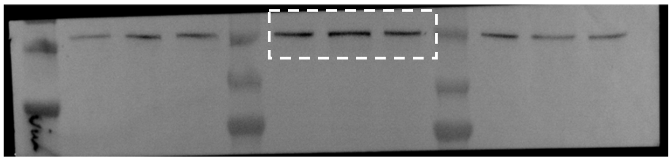

Fig. 3E, Vinculin

Uncropped images in Figure 4

Fig. 4C, Aggregated mHTT

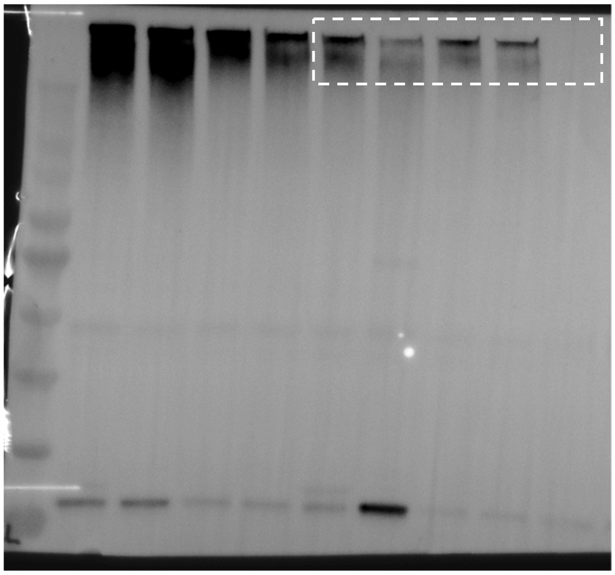

Fig. 4C, HA

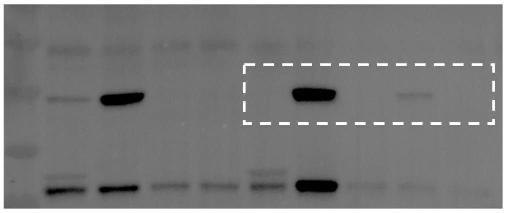

Fig. 4C, Vinculin

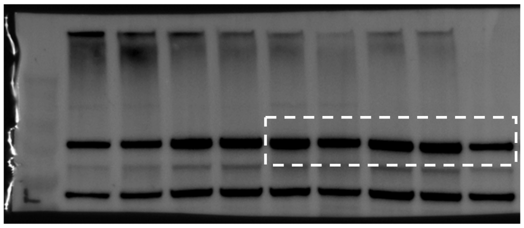

Fig. 4C, Full length mHTT

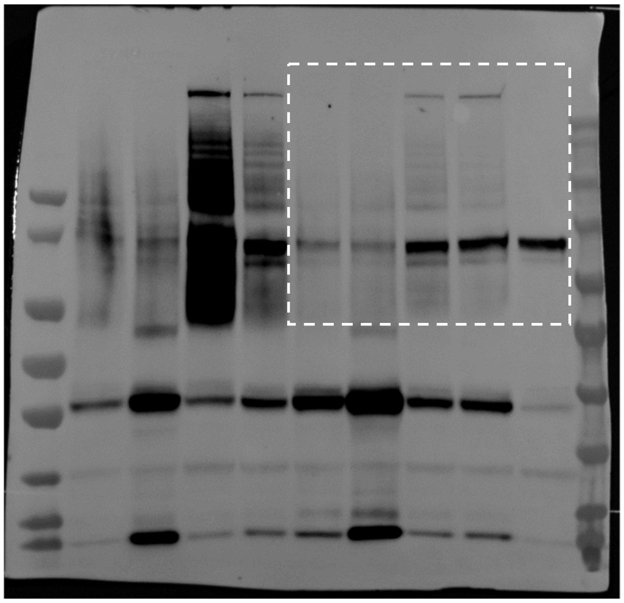

Uncropped images in Figure 5

Fig. 5B, HTT

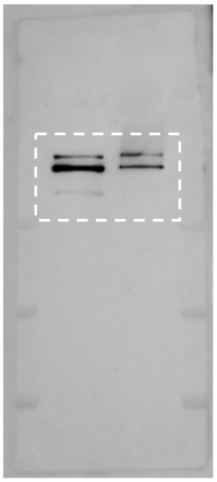

Fig. 5A, HTT

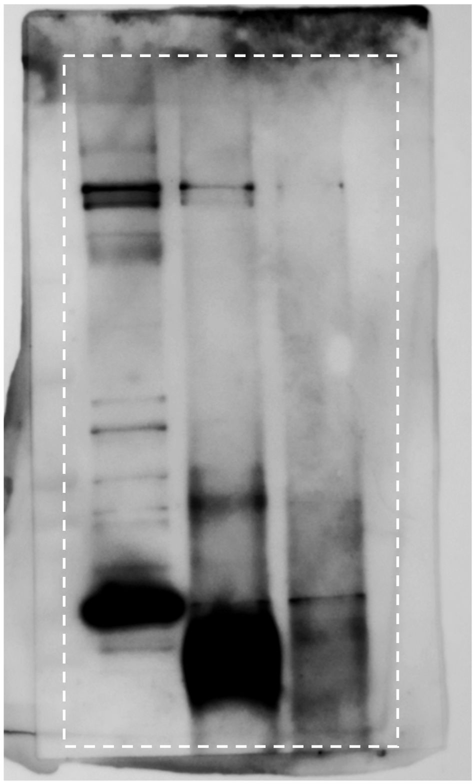

Fig. 5D, HTT

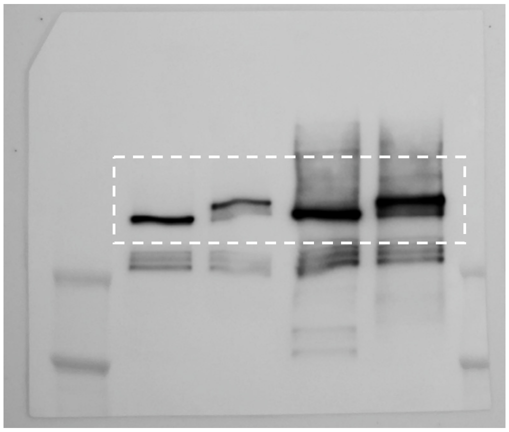

Fig. 5B, HAP40

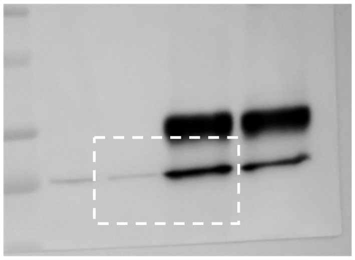

Fig. 5D, HAP40

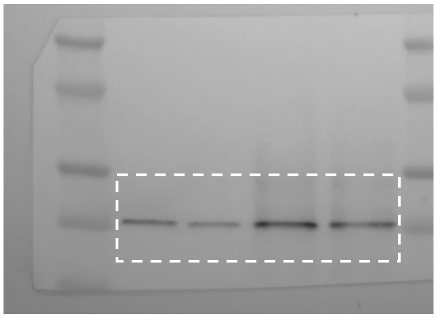

Fig. 5A, HAP40

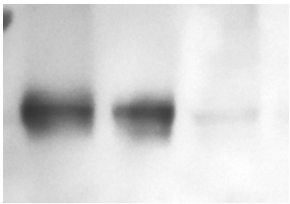

Fig. 5G, Exon 1

Fig. 5G 212aa

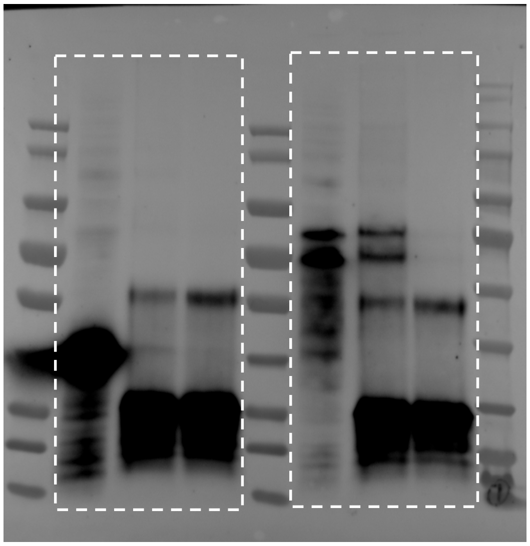

Fig. 5G, 927aa

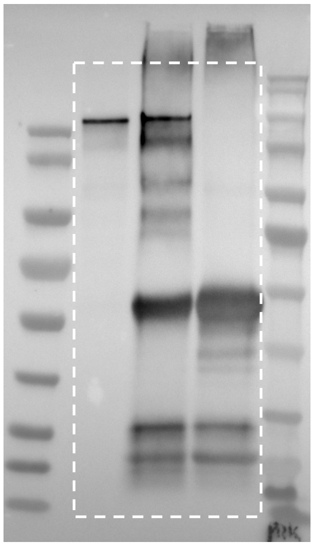

Fig. 5G, HAP40

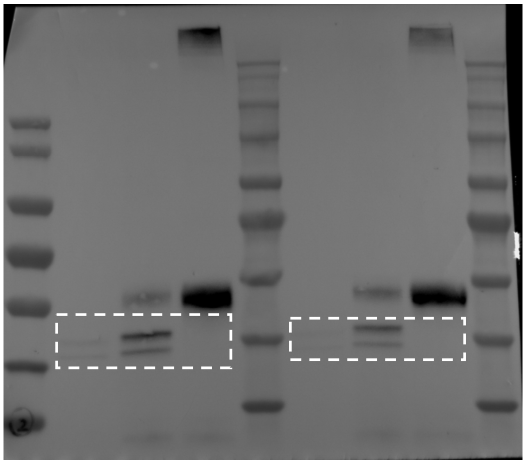

Fig. 5G, HAP40

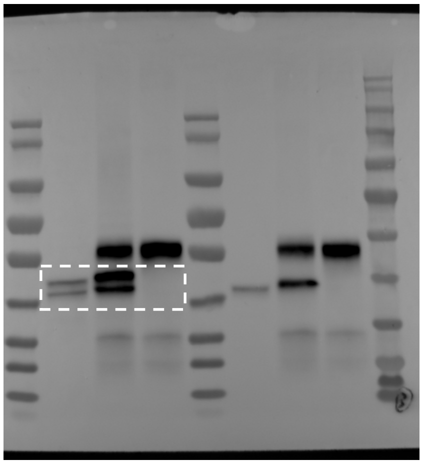

Uncropped images in Figure 6

Fig. 6A, Total ubiquitin

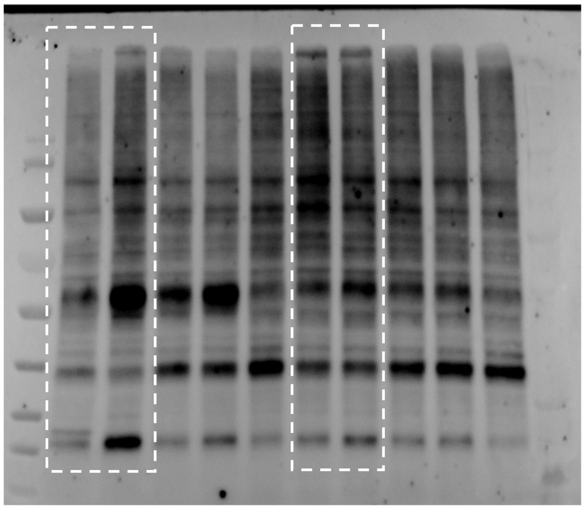

Fig. 6A, Rab5a

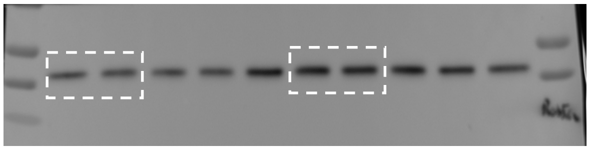

Fig. 6A, EEA1

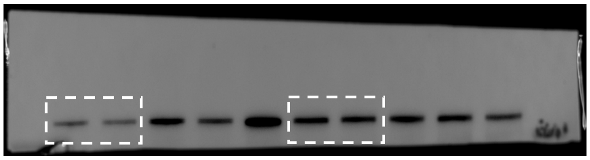

Fig. 6A, Vinculin

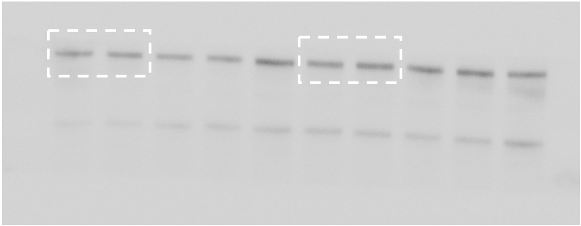

Fig. 6A, LC3

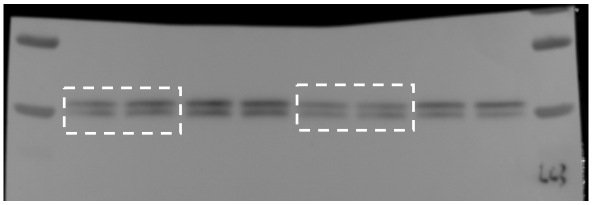

Fig. 6C, 23Q-HEK293, K48 ubiquitin

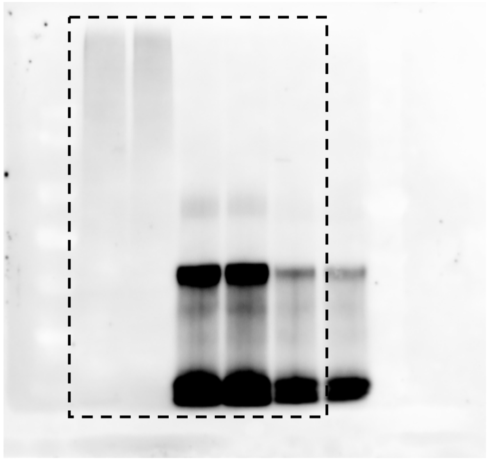

Fig. 6C, 23Q-HEK293, HTT

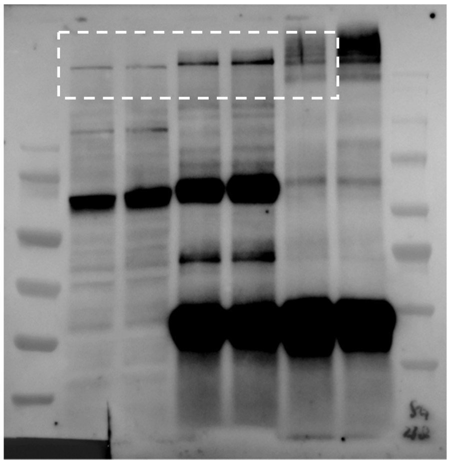

Fig. 6C, 120Q-HEK293, K48 ubiquitin

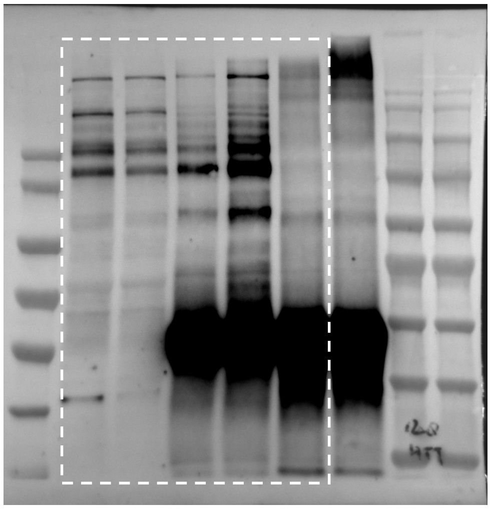

Fig. 6C, 120Q-HEK293, HTT

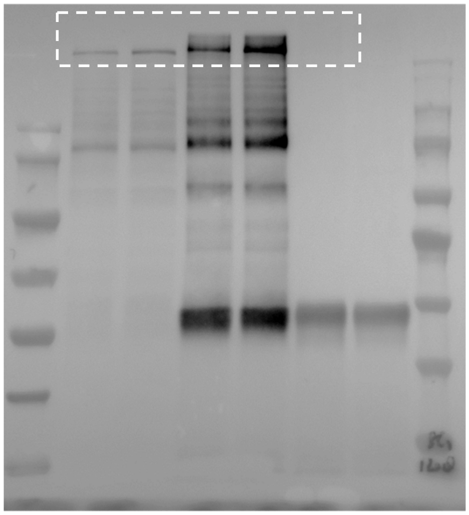

Uncropped images in Figure S1

Fig. S1A, HTT

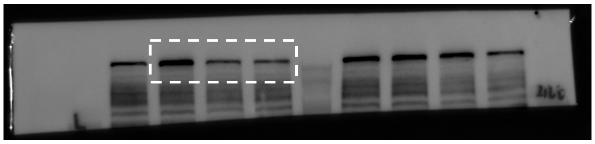

Fig. S1A, HAP40

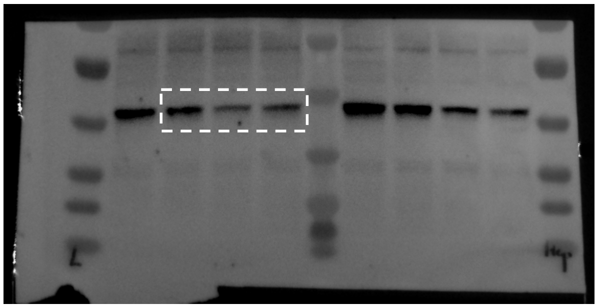

Fig. S1A, Vinculin

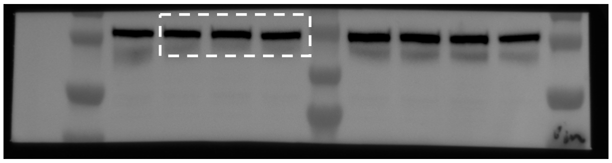

Fig. S1C, HTT

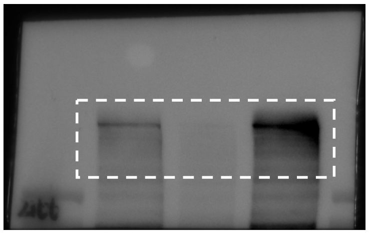

Fig. S1C, HAP40

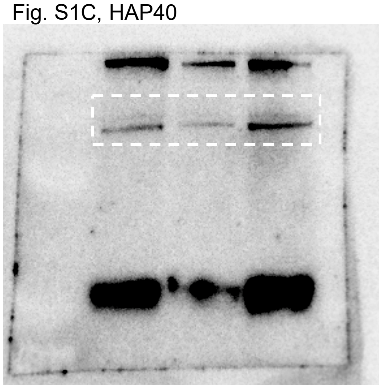

Fig. S1C, Histone H3

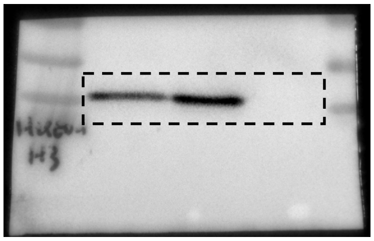

Fig. S1C, GAPDH

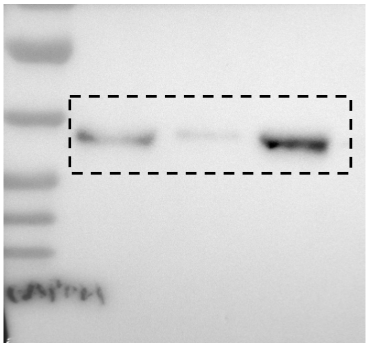

Fig. S2B, HTT

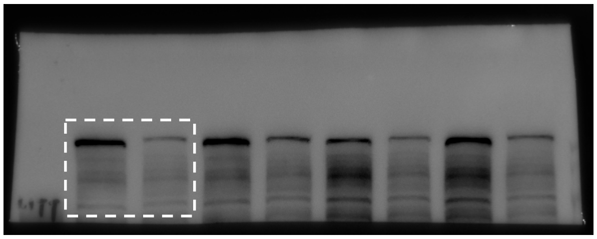

Fig. S2B, HAP40

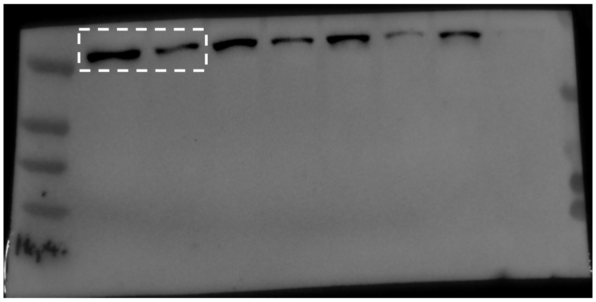

Fig. S2B, GFAP

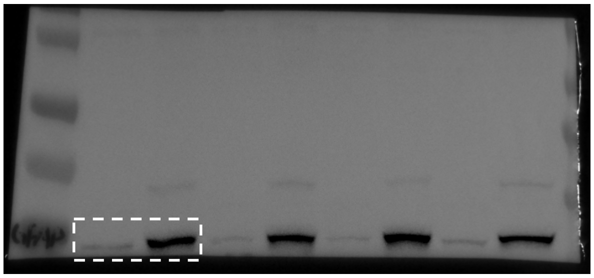

Fig. S2B, NeuN

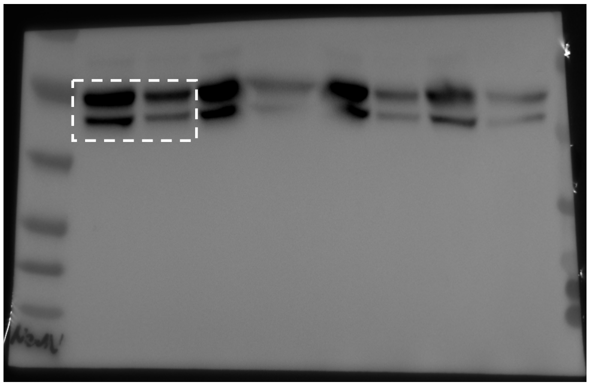

Fig. S4A

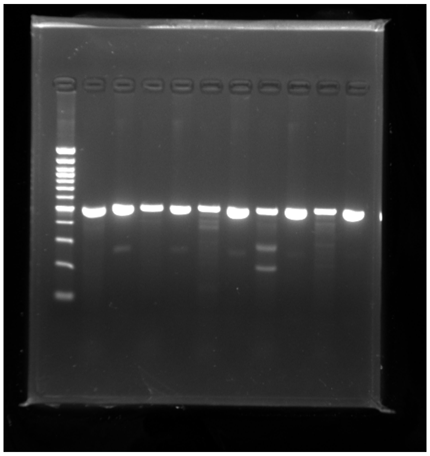

Fig. S4B, HTT

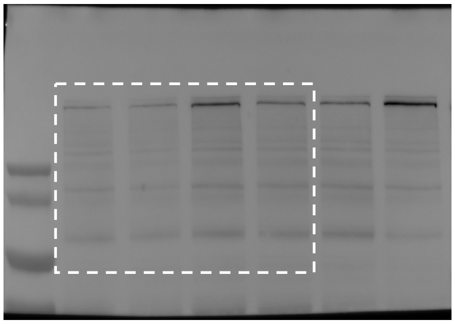

Fig, S4B, Vinculin

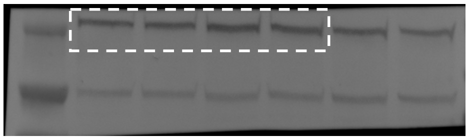

Fig. S4B, HAP40

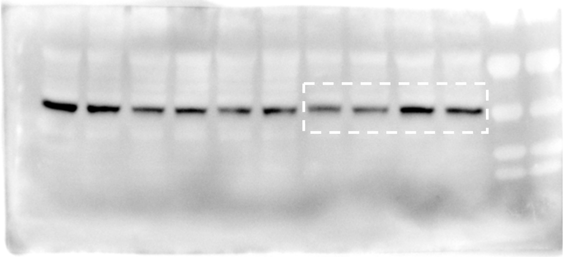

Fig. S6A, HTT

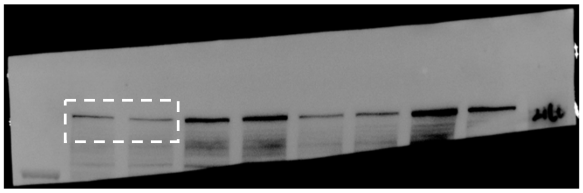

Fig. S6A, Cas9

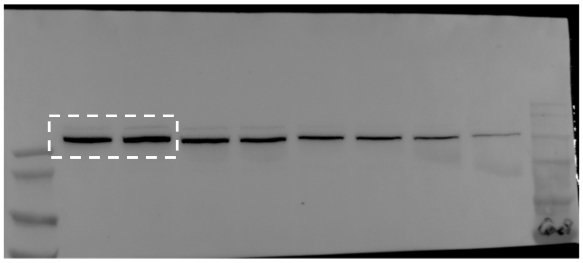

Fig. S6A, RFP

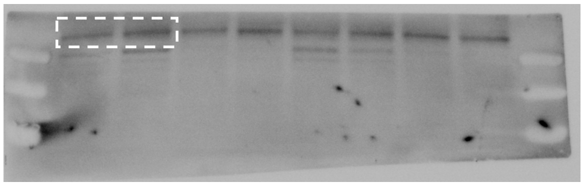

Fig. S6A, Vinculin

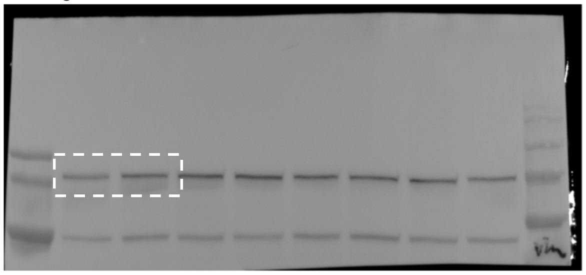

Fig. S6A, HAP40

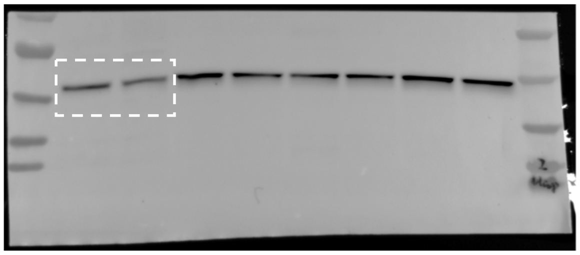

Fig. S6A, NeuN

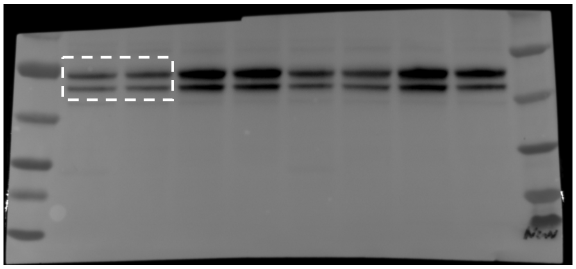

Fig. S6A, GFAP

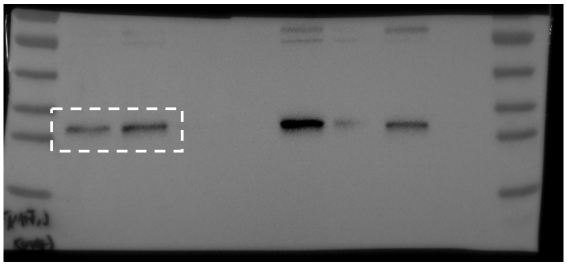

Uncropped images in Figure S7

Fig. S7D, HAP40

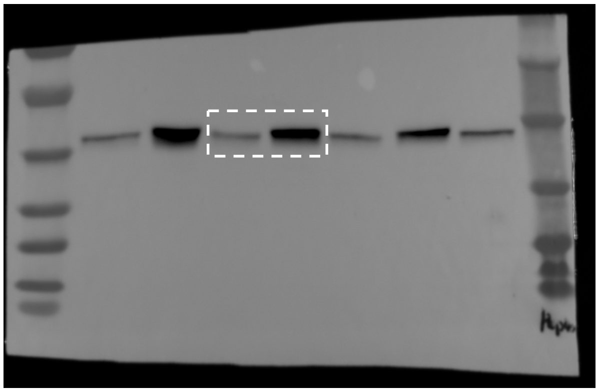

Fig. S7D, NeuN

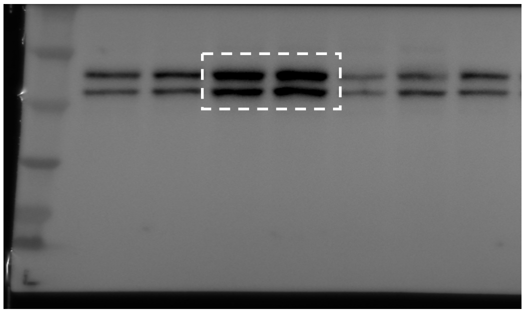

Fig. S7D, Vinculin

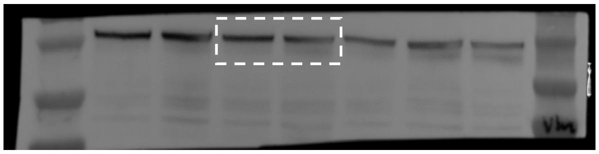

Fig. S7D, HTT (MAB2166)

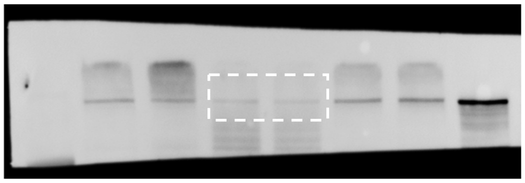

Fig. S8A, HAP40

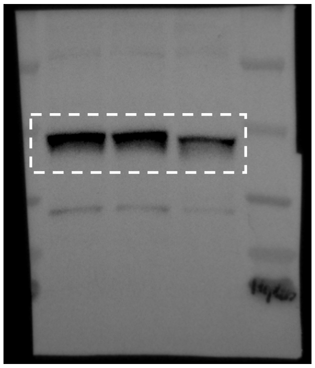

Fig. S8A, GFP

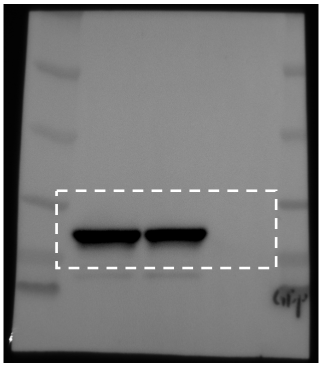

Fig. S8A, Vinculin

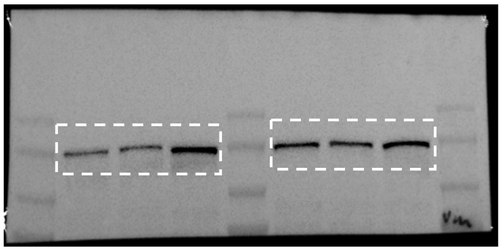

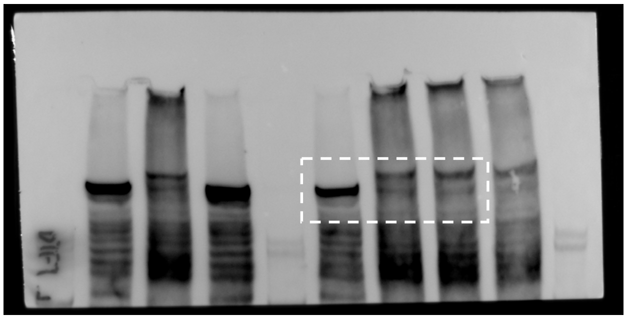

Fig. S8B, HTT

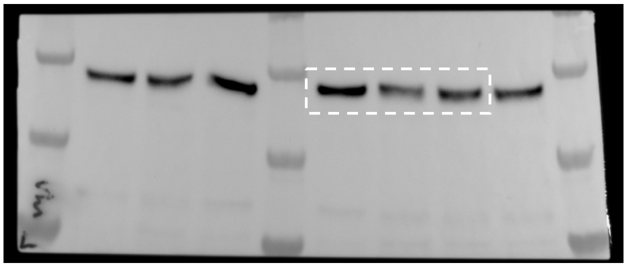

Fig. S8B, Vinculin

Uncropped images in Figure S9

Fig. S9A, Cas9

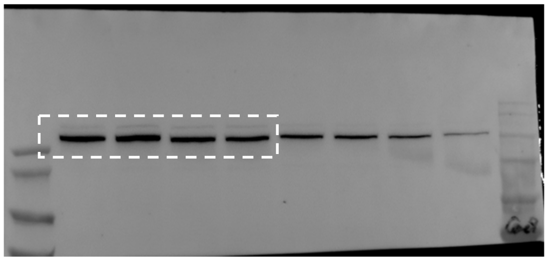

Fig. S9A, HAP40

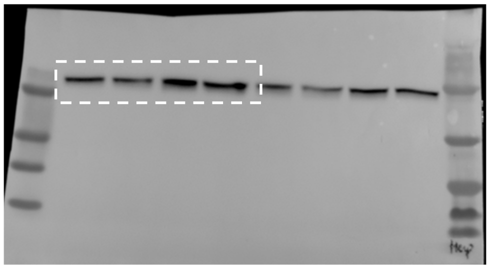

Fig. S9A, Vinculin

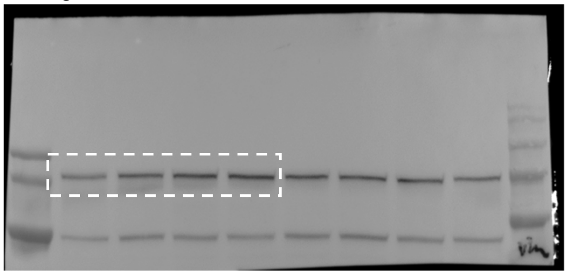

Fig. S9A, RFP

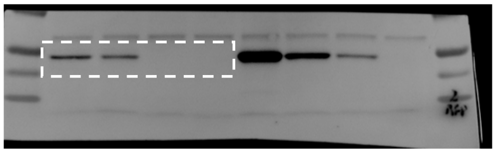

Fig. S9A, Total ubiquitin

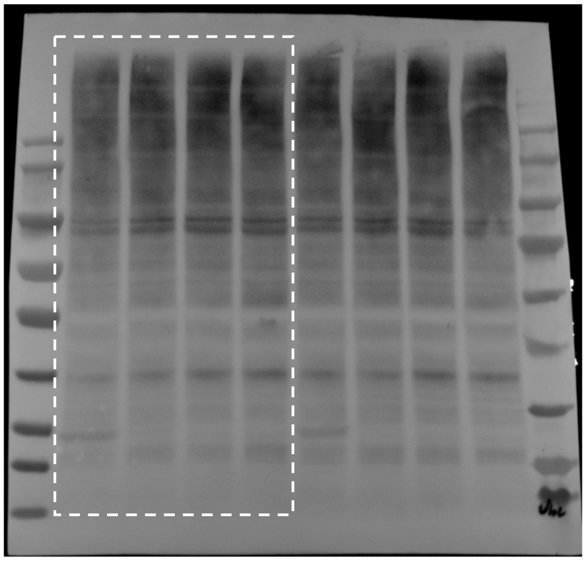

Fig. S9C, HTT

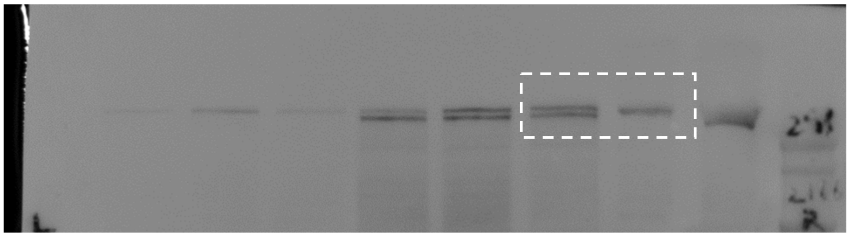

Fig. S9C, Vinculin

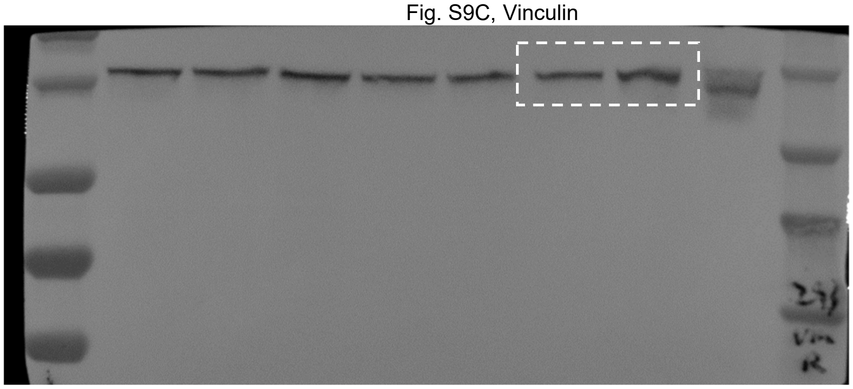

Fig.S9D, HAP40

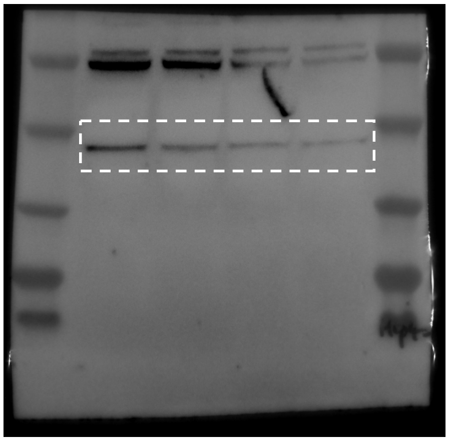

Fig. S9D, Vinculin

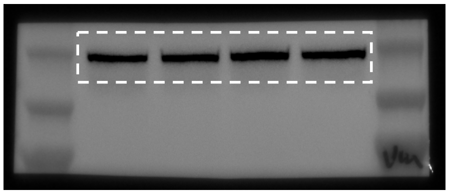

Fig. S9E, K48 Ubiquitin

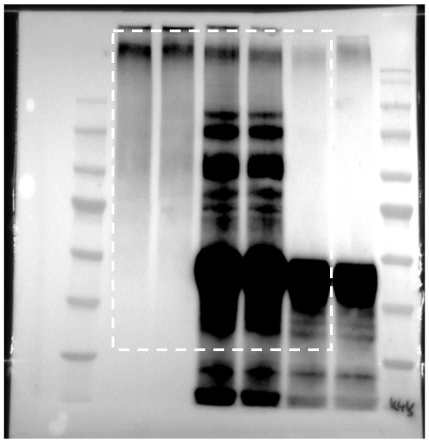

Fig. S9E, HTT

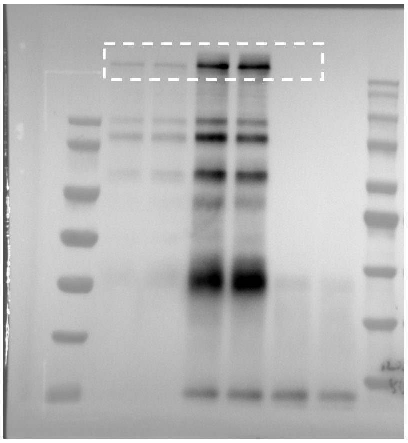

Supplement: Supplementary file 2 — Full and uncropped western blots [file 41419_2024_6716_MOESM2_ESM.pdf]
